# Multi-marker testing based on accelerated failure time models under possible left truncation and competing risks

**DOI:** 10.1093/bib/bbag155

**Published:** 2026-04-08

**Authors:** Chenxi Li, Di Wu, Qing Lu

**Affiliations:** Department of Epidemiology and Biostatistics, Michigan State University, 909 Wilson Road, 48824 MI, United States; Department of Epidemiology and Biostatistics, Michigan State University, 909 Wilson Road, 48824 MI, United States; Department of Biostatistics, University of Florida, 2004 Mowry Road, 32611 FL, United States

**Keywords:** accelerated failure time model, competing risks, genetic heterogeneity, kernel functions, left truncation, multi-marker tests

## Abstract

Kernel-based multi-marker tests for survival outcomes use primarily the Cox model to adjust for covariates. The proportional hazards assumption made by the Cox model could be unrealistic, especially in the long-term follow-up. We develop a suite of novel multi-marker survival tests for genetic association and interaction based on the accelerated failure time model, which is a popular alternative to the Cox model due to its direct physical interpretation. The tests are based on the asymptotic distributions of their test statistics and are thus computationally efficient. The association tests can account for the heterogeneity of genetic effects across subpopulations/individuals to increase the power. All the new tests can deal with competing risks and left truncation. Moreover, we develop small-sample corrections to the tests to improve their accuracy under small samples. Extensive numerical experiments show that the new tests perform very well in various scenarios. An application to a genetic dataset of Alzheimer’s disease illustrates the tests’ practical utility.

## Introduction

Multi-marker tests have been popular for genome-wide association studies (GWAS) and transcriptomic profiling since the seminal paper on sequence kernel association test (SKAT) by Wu *et al.* [1] was published. By testing the joint effect of genetic markers in a knowledge-based region (e.g. a gene region or a biological pathway), multi-marker tests aggregate the association signals and reduce the multiple testing burden as opposed to single-marker tests, thereby improving the power for association discovery. In addition, most multi-marker tests are kernel-based, which account for inter-marker correlations and thus have higher power compared with the regular tests for testing multiple markers, e.g. F-tests and likelihood-based tests (i.e. Wald, score, and likelihood ratio tests) [2].

Although there has been a rich literature of multi-marker tests for quantitative and binary traits, e.g. Wu *et al.* [1], Lee *et al.* [3], and Ionita-Laza *et al.* [4] to name a few, the field of multi-marker tests for censored survival outcomes is far less developed, primarily in four aspects. First, the types of covariate-adjustment models used by the existing multi-marker survival tests are limited. Most of the existing tests are based on the Cox model, including Goeman *et al.* [5], Cai *et al.* [6], Chen *et al.* [7], and Li *et al.* [8]. Only three works used non-Cox models, which are Sinnott and Cai [9] for the accelerated failure time (AFT) model and Tzeng *et al.* [10] and Wu *et al.* [11] for linear transformation models. Misspecifying the covariate-adjustment model will lead to an incorrect null distribution for a multi-marker test (as illustrated in Supplementary Fig. 6), hampering the gene discovery process. Second, all the existing multi-marker survival tests apply only to time-to-event outcomes, while other types of survival phenotypes, e.g. competing risks and recurrent events, are not uncommon in genetic studies of human diseases. Third, all the existing tests except Goeman *et al.* [5], Chen *et al.* [7], and Tzeng *et al.* [10] are not valid in the general situation where the adjustment covariates are correlated with the genetic markers under testing; see Li *et al.* [8] for relevant discussion and simulations. The tests of Li *et al.* [8] can only adjust for linear confounding, namely the genetic markers are linearly correlated with the confounders. Fourth, many existing tests, including Goeman *et al.* [5], Sinnott and Cai [9], and Chen *et al.* [7], are not accurate in terms of the null distribution of p-value under small or even modest sample sizes, as shown by Li *et al.* [8].

In this article, we propose a set of multi-marker tests for survival outcomes based on the AFT model, a popular alternative to the Cox model in survival analysis due to its direct physical interpretation [12]. They can be used to test genetic associations and gene–gene/gene–environment interactions. In testing associations, the proposed association tests can account for possible genetic heterogeneity (i.e. the genetic effect varies across subpopulations or individuals) to improve power. Compared with the existing AFT multi-marker test [9], besides being able to account for genetic heterogeneity, our tests use analytic null distributions to compute the p-values, can deal with competing risks and left truncation, can adjust for confounding regardless of the relationship between the markers and the confounders, and most importantly are much more accurate under small and modest sample sizes.

Our methods were motivated by the data from the Rush Memory and Aging Project and the Religious Orders Study (ROSMAP) [13]. These two studies are both ongoing cohort studies of aging and Alzheimer’s disease (AD). Both studies have over 20 years of follow-up and together generated genome-wide data for over 1600 subjects. So the ROSMAP data are a great resource for studying genetic risk factors for incident AD. Nonetheless, the Cox model may not fit the time-to-AD data of ROSMAP given the long follow-up, and the genetic analysis of time-to-AD data needs to account for the competing risk of death without AD and the left truncation of survival outcome if the time scale is age. These considerations motivated us to develop the methods of this paper.

The rest of the paper is organized as follows. The Methods section describes the proposed tests. The proof of their asymptotic null distributions is deferred to Supplementary Appendix A. The Numerical Methods section shows extensive simulations to evaluate the finite-sample performance of the methods. The section titled ‘A real application’ presents an application of the new tests to a gene-based association analysis of age at AD onset with the ROSMAP data. The paper concludes with some discussion on future research directions in the Discussion section.

## Methods

We develop new genetic association and interaction tests just for competing risks data under left truncation, since regular survival data with/without left truncation will be special cases where there is only one failure cause.

### Association tests

Consider a cohort study where participants have not experienced any competing risk events at baseline. The sample size is denoted by $n$. The observed survival data of a subject are $(A,\widetilde{T},\Delta ,\epsilon \Delta )$, where $\widetilde{T}={\mathrm{min}} (T,C)$, $T$ is the time to failure, $C$ is the time from the time origin for $T$ (the time point at which $T=0$) to censoring, $\Delta =I(T\le C)$, $\epsilon $ is the failure cause, and $A$ is the left truncation time, namely the time from the time origin for $T$ to the time point when the subject enters the follow-up, e.g. age at study entry in an analysis of age at onset of a disease. Suppose that there are $J$ failure causes, denoted by $1,\ldots ,J$. We are interested in testing the effect of a set of genetic markers ${\mathbf G}\equiv (G_{1},\ldots ,G_{p})^{T}$, e.g. in a gene or biological pathway, on Cause 1, where $G_{i}$ denotes the value of the $i$th marker $(i=1,\ldots ,p)$, e.g. the number of minor alleles at the $i$th SNP. We develop two association tests to accomplish this objective. One of the tests considers the possibility that the effect of ${\mathbf G}$ varies across individuals or certain subpopulations, e.g. different genome profiles, sexes, or races. The population structure is either explicit, which can be indicated by a vector of observable variables, ${\mathbf X}\equiv (X_{1},\ldots ,X_{D})^{T}$ (e.g. race), or latent (e.g. subpopulations with different ancestry backgrounds) but inferable by a vector of observable variables, also denoted by ${\mathbf X}$ (e.g. a large number of SNPs from GWAS data). The other new association test does not consider genetic heterogeneity. In both tests, we adjust for covariates ${\mathbf Z}\equiv (Z_{1},\ldots , Z_{q})^{T}$ to reduce confounding and/or increase power. ${\mathbf X}$ is part of ${\mathbf Z}$ when the subpopulations are explicit. We assume that the dyad of failure time and cause, the truncation time, and the residual censoring time $C-A$ are conditionally independent given ${\mathbf G}$ and ${\mathbf Z}$ (and ${\mathbf X}$ if considering genetic heterogeneity).

The common null hypothesis of our association tests is that ${\mathbf G}$ has no effect on the cause-specific hazard (CSH) of Cause 1 after adjusting for ${\mathbf Z}$ (in any subpopulation if considering genetic heterogeneity). We assume that, under the null, the CSH of Cause 1 given ${\mathbf Z}$ follows an AFT model:


(1) \begin{align*}& \lambda(t;{\mathbf Z})=\lambda_{0}(te^{-{\boldsymbol{\beta}}^{T}{\mathbf Z}})e^{-{\boldsymbol{\beta}}^{T}{\mathbf Z}},\end{align*}

where $\lambda _{0}(\cdot )$ is an unspecified baseline CSH.

**Figure 1:**
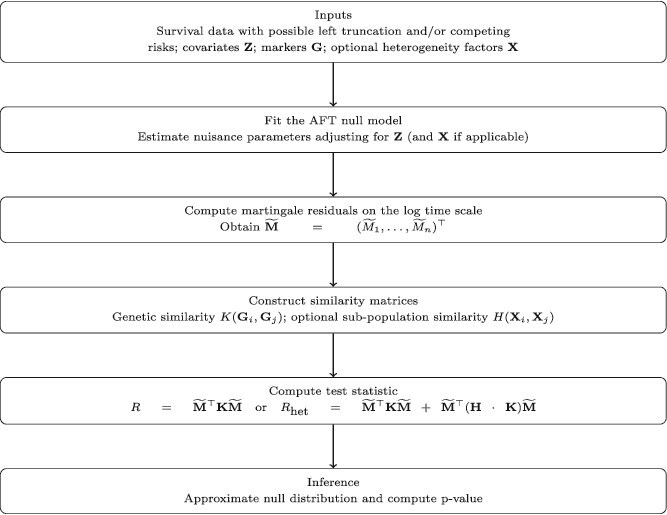
Overview of the proposed association testing framework.

The two proposed association tests share a general testing framework that is outlined in Fig. 1. The steps are detailed below.

The association tests involve fitting the null model (1) to the data at first. Specifically, we estimate ${\boldsymbol{\beta }}$ and $\Lambda _{0}(\cdot )\equiv \int _{0}^\cdot \lambda _{0}(s)ds$ by applying the rank-based estimation method [14] to a (working) AFT model:


(2) \begin{align*}& {\mathrm{log}}(T^{*})={\boldsymbol{\beta}}^{T}{\mathbf Z}+\varepsilon.\end{align*}

The (working) response $T^{*}$ is subject to left truncation with truncation time being $A$, which equals $\widetilde{T}$ if $\epsilon \Delta =1$, and is right censored at $\widetilde{T}$ otherwise. The random error $\varepsilon $ is independent of ${\mathbf Z}$, and the hazard function of $\varepsilon $ is $\lambda _{\varepsilon }(s)=e^{s}\lambda _{0}(e^{s})$. Following Chiou and Xu [14], we define $e^{a}_{i}({\boldsymbol{\beta }})={\mathrm{log}} A_{i}-{\boldsymbol{\beta }}^{T}{\mathbf Z}_{i}$, $e_{i}({\boldsymbol{\beta }})={\mathrm{log}} \widetilde{T}_{i}-{\boldsymbol{\beta }}^{T}{\mathbf Z}_{i}$, $N_{i}({\boldsymbol{\beta }},t)=I(e_{i}({\boldsymbol{\beta }})\le t, \epsilon \Delta =1)$, $Y_{i}({\boldsymbol{\beta }},t)=I(e_{i}({\boldsymbol{\beta }})\ge t)$, $\nu _{i}({\boldsymbol{\beta }},t)=I(e^{a}_{i}({\boldsymbol{\beta }})< t)$. The quantities $N_{i}({\boldsymbol{\beta }},t)$ and $\nu _{i}({\boldsymbol{\beta }},t)Y_{i}({\boldsymbol{\beta }},t)$ are, respectively, the counting and the at-risk processes of subject $i$ on the transformed time scale of ${\mathrm{log}} (T^{*})-{\boldsymbol{\beta }}^{T}{\mathbf Z}$. Given ${\boldsymbol{\beta }}$, $\Lambda _\varepsilon (t)\equiv \int _{-\infty }^{t}\lambda _\varepsilon (s)ds$ is estimated by a Nelson–Aalen type estimator [14],


(3) \begin{align*}&\widehat{\Lambda}_\varepsilon({\boldsymbol{\beta}},t)=\int_{-\infty}^{t}\left\{\sum_{i=1}^{n}\nu_{i}({\boldsymbol{\beta}},s)Y_{i}({\boldsymbol{\beta}},s)\right\}^{-1}\sum_{i=1}^{n}dN_{i}({\boldsymbol{\beta}},s).\end{align*}

The regression coefficients, ${\boldsymbol{\beta }}$, are estimated from the rank-based estimating equation, Equation (5) in Chiou and Xu [14], with log-rank weights. The resulting estimator is denoted by $\widehat{{\boldsymbol{\beta }}}$.

Define $M_{i}(\Lambda _\varepsilon ,{\boldsymbol{\beta }})=N_{i}({\boldsymbol{\beta }},\infty )-\int _{-\infty }^\infty \nu _{i}({\boldsymbol{\beta }},t)Y_{i}({\boldsymbol{\beta }},t)d\Lambda _\varepsilon (t)$, $M_{i}=M_{i}(\Lambda _\varepsilon ,{\boldsymbol{\beta }})$, $\widehat{M}_{i}=M_{i}(\widehat{\Lambda }_\varepsilon ({\boldsymbol{\beta }},\cdot ),{\boldsymbol{\beta }})$, and $\widetilde{M}_{i}=M_{i}(\widehat{\Lambda }_\varepsilon (\widehat{{\boldsymbol{\beta }}},\cdot ),\widehat{{\boldsymbol{\beta }}})$. The quantity $\widetilde{M}_{i}$ can be viewed as a martingale residual on the transformed time scale of ${\mathrm{log}} (T^{*})-{\boldsymbol{\beta }}^{T}{\mathbf Z}$ since the process $M_{i}(t)=N_{i}({\boldsymbol{\beta }},t)-\int _{-\infty }^{t}\nu _{i}({\boldsymbol{\beta }},u)Y_{i}({\boldsymbol{\beta }},u)d\Lambda _\varepsilon (u)$ is a martingale when the null hypothesis of no association is true. The proposed association test that does not consider genetic heterogeneity is based on the test statistic,


(4) \begin{align*}& R\equiv\widetilde{{\mathbf M}}^{T}{\mathbf K}\widetilde{{\mathbf M}},\end{align*}

where $\widetilde{{\mathbf M}}=(\widetilde{M}_{1},\ldots ,\widetilde{M}_{n})^{T}$, ${\mathbf K}=\{K({\mathbf G}_{i},{\mathbf G}_{j})\}_{n\times n}$, and $K(\cdot ,\cdot )$ is a Mercer kernel function (Herbrich [15], p. 35). Note that


(5) \begin{align*}& R=\sum_{i=1}^{n}\sum_{j=1}^{n} K({\mathbf G}_{i},{\mathbf G}_{j})\widetilde{M}_{i}\widetilde{M}_{j}.\end{align*}

The quantity $\widetilde{M}_{i}\widetilde{M}_{j}$ can be viewed as a concordance measure of phenotypic deviations from the null model between subjects $i$ and $j$. The quantity $K({\mathbf G}_{i},{\mathbf G}_{j})$ equals the cross product of ${\boldsymbol{\phi }}({\mathbf G}_{i})$ and ${\boldsymbol{\phi }}({\mathbf G}_{j})$, the features obtained by mapping ${\mathbf G}_{i}$ and ${\mathbf G}_{j}$, respectively, to the feature space induced by $K(\cdot ,\cdot )$ (Herbrich [15], Chap. 2), and thus can be viewed as a genetic similarity between subjects $i$ and $j$. Under the null, the residual $\widetilde{M}_{i}$ tends to have smaller magnitude than under the alternative hypothesis, and it satisfies $E(\widetilde{M}_{i}|{\mathbf G}_{i})\approx 0$, which implies $E[\widetilde{M}_{i}\widetilde{M}_{j}K({\mathbf G}_{i},{\mathbf G}_{j})]\approx 0$ under the null. In contrast, under the alternative, subjects who are genetically more similar (i.e. with larger $K({\mathbf G}_{i},{\mathbf G}_{j})$) tend to deviate from the null model in the same direction, so that $\widetilde{M}_{i}\widetilde{M}_{j}$ is more often positive for such pairs. As a result, $E[\widetilde{M}_{i}\widetilde{M}_{j}K({\mathbf G}_{i},{\mathbf G}_{j})]$ becomes substantially larger than zero under the alternative. These considerations imply that the test statistic $R$ is expected to take larger values under the alternative than under the null.

The choice of $K(\cdot ,\cdot )$ depends on the expected effect form of ${\mathbf G}$ in a way that the induced feature space by $K(\cdot ,\cdot )$ should match that form. For example, if the effect of ${\mathbf G}$ is expected to be linear, we use the linear kernel, a.k.a. cross-product kernel, $K({\mathbf G}_{i},{\mathbf G}_{j})={\mathbf G}_{i}^{T}{\mathbf G}_{j}$, for which the induced feature space is the input space $\{{\mathbf G}\}$. If ${\mathbf G}$ is a vector of SNP covariates, each of which is expected to have a nonlinear effect, we use the IBS kernel, $K({\mathbf G}_{i},{\mathbf G}_{j})=\sum _{k=1}^{p}(2-|G_{i,k}-G_{j,k}|)/(2p)$. If ${\mathbf G}$ is a vector of gene expression covariates that are expected to have nonlinear and/or interactive effects, we use a polynomial kernel, $K({\mathbf G}_{i},{\mathbf G}_{j})=(\rho +{\mathbf G}_{i}^{T}{\mathbf G}_{j})^{d}$, or the Gaussian kernel, $K({\mathbf G}_{i},{\mathbf G}_{j})=\exp (-\rho \|{\mathbf G}_{i}-{\mathbf G}_{j}\|^{2})$, where $d$ is a specified positive integer and $\rho $ is a specified positive constant. As suggested by Wei and Lu [16], a universal genetic similarity kernel could be the Laplacian kernel, $K({\mathbf G}_{i},{\mathbf G}_{j})=\exp (-\sum _{k=1}^{p}w_{k}|G_{ik}-G_{jk}|/\Upsilon )$, where $G_{ik}$ can be discrete or continuous variables, $\Upsilon =\sum _{k=1}^{p}w_{k} $, and $w_{k}$ is the reciprocal of the sample standard deviation of $G_{k}$. This kernel is particularly useful for association mapping from sequencing reads, which involves many rare variants.

Suppose that the rank of ${\mathbf K}$ is $m$. Then ${\mathbf K}={\mathbf E}_{1}^{T}{\mathbf E}_{1}$ for some $m\times n$ matrix ${\mathbf E}_{1}$. In Supplementary Appendix A, we derive an estimator of $Cov({\mathbf E}_{1}\widetilde{{\mathbf M}})$ under the null hypothesis and a large sample size. Denote the eigenvalues of this covariance matrix estimator by $\lambda _{1},\ldots ,\lambda _{m}$. It is shown in Supplementary Appendix A that the large-sample null distribution of $R$ is approximately $\sum _{j=1}^{m}\lambda _{j}\chi _{1j}^{2},$ where $\chi _{1j}^{2}$’s are independent chi-square variables with degree 1. Based on this distribution, we compute the p-value, $P(R\ge R_{\mbox{obs}})$, using Davies’ method [17].

The proposed association test that considers genetic heterogeneity is based on the test statistic,


(6) \begin{align*}& R_{\mbox{het}}\equiv\widetilde{{\mathbf M}}^{T}{\mathbf W}\widetilde{{\mathbf M}},\end{align*}

where ${\mathbf W}=({\mathbf J}+{\mathbf H})\cdot{\mathbf K}$, ${\mathbf J}=\{1\}_{n\times n}$, ${\mathbf H}=\{H({\mathbf X}_{i},{\mathbf X}_{j})\}_{n\times n}$, $H(\cdot ,\cdot )$ is a kernel function measuring the subpopulation similarity, and $\cdot $ represents the Hadamard product. The quantity ${\mathbf W}$ can be viewed as a heterogeneity-weighted genetic similarity matrix. Note that $R_{\mbox{het}}=\widetilde{{\mathbf M}}^{T} {\mathbf K}\widetilde{{\mathbf M}}+\widetilde{{\mathbf M}}^{T}({\mathbf H}\cdot{\mathbf K})\widetilde{{\mathbf M}}$. Thus, it can be thought to simultaneously test the main effect of ${\mathbf G}$ (through $\widetilde{{\mathbf M}}^{T} {\mathbf K}\widetilde{{\mathbf M}}$) and its interaction with the subpopulation (through $\widetilde{{\mathbf M}}^{T} ({\mathbf H}\cdot{\mathbf K})\widetilde{{\mathbf M}}$).

The choice of $H(\cdot ,\cdot )$ depends on the type of ${\mathbf X}$. If ${\mathbf X}$ is a set of dummy variables coding explicit subpopulations, e.g. different sexes, we can use the identity kernel $H({\mathbf X}_{i},{\mathbf X}_{j})=I({\mathbf X}_{i}={\mathbf X}_{j})$. If ${\mathbf X}$ is a set of SNPs, we can choose the IBS kernel for $H({\mathbf X}_{i},{\mathbf X}_{j})$. If ${\mathbf X}$ is a set of continuous variables, the Gaussian kernel can be used.

The approximate null distribution of $R_{\mbox{het}}$ can be derived the same way as for $R$, except that ${\mathbf K}$ is replaced by ${\mathbf W}$ in the derivation. Based on this distribution, we compute the p-value, $P(R_{\mbox{het}}\ge R_{\mbox{het}}^{\mbox{obs}})$, using Davies’ method [17].

The derivations of the large sample null distributions of $R$ and $R_{\mbox{het}}$ do not require any assumption about the relationship between ${\mathbf G}$ and ${\mathbf Z}$. Therefore, the two association tests can adjust for confounding regardless of the relationship between the markers and the confounders. This is a desirable property for (epi)genetic association tests and differential expression tests, because confounding is ubiquitous in those data analyses and usually has an unknown form. Some early multi-marker survival tests cannot adjust for any confounding, e.g. Cai *et al.* [6] and Sinnott and Cai [9], as shown in Li *et al.* [8]. The multi-marker survival tests proposed by Li *et al.* [8] can only adjust for linear confounding, namely, ${\mathbf G}={\mathbf a}+{\mathbf B}^{T}{\mathbf Z}_{c}+{\mathbf e}$, where ${\mathbf Z}_{c}$ is a vector of confounders, ${\mathbf a}$ and ${\mathbf B}$ are, respectively, a constant vector and a constant matrix, and ${\mathbf e}$ is a zero-mean random error vector,i.e. independent of ${\mathbf Z}_{c}$. In genetic association analyses, the markers and the confounders are usually minor allele counts of SNPs and the top few principal components of the genome-wide genotype data, respectively, and so the above linear model between ${\mathbf G}$ and ${\mathbf Z}_{c}$ cannot hold because the conditional variance of ${\mathbf G}$ given ${\mathbf Z}_{c}$ depends on the conditional mean. In differential gene expression analyses, confounding usually does not have the above linear form either. For example, ${\mathbf Z}_{c}$ often includes age of the subject, and there is evidence that age affects both mean and variance of gene expression [18].

### Interaction tests

The set-up for our gene–environment (G-E) interaction tests is the same as that for the association tests, except that ${\mathbf X}$ is a set of environmental covariates and is included in ${\mathbf Z}$. We aim to test whether ${\mathbf G}$ and ${\mathbf X}$ have an interaction effect on Failure Cause 1. The common null hypothesis of our G-E interaction tests is that ${\mathbf G}$’s effect on the CSH of Cause 1 adjusted for ${\mathbf Z}$ does not vary with ${\mathbf X}$.

Our interaction tests differ according to the expected effect form of ${\mathbf G}$ under the null. If ${\mathbf G}$ is expected to have a linear effect, we assume that, under the null, the CSH of Cause 1 given ${\mathbf G}$ and ${\mathbf Z}$ follows an AFT model:


(7) \begin{align*}& \lambda(t;{\mathbf G},{\mathbf Z})=\lambda_{0}(te^{-{\boldsymbol{\alpha}}^{T}{\mathbf G}-{\boldsymbol{\beta}}^{T}{\mathbf Z}})e^{-{\boldsymbol{\alpha}}^{T}{\mathbf G}-{\boldsymbol{\beta}}^{T}{\mathbf Z}}.\end{align*}

If ${\mathbf G}$ is a vector of SNPs, and the mode of inheritance for each SNP in ${\mathbf G}$ is unspecified, we assume that, under the null, the CSH of Cause 1 given ${\mathbf G}$ and ${\mathbf Z}$ follows another AFT model:


(8) \begin{align*}\lambda(t;{\mathbf G},{\mathbf Z})=&\lambda_{0}(te^{-\sum_{m=1}^{p}\{I(G_{m}=1)\alpha_{1m}+I(G_{m}=2)\alpha_{2m}\}-{\boldsymbol{\beta}}^{T}{\mathbf Z}}) \nonumber \\& e^{-\sum_{m=1}^{p}\{I(G_{m}=1)\alpha_{1m}+I(G_{m}=2)\alpha_{2m}\}-{\boldsymbol{\beta}}^{T}{\mathbf Z}}.\end{align*}

Regardless of which AFT null model is assumed, the interaction tests involve fitting the null model first, using the rank-based estimation method with log-rank weights [14]. Denote the resulting estimators for ${\boldsymbol{\alpha }}$ and ${\boldsymbol{\beta }}$ by $\widehat{{\boldsymbol{\alpha }}}$ and $\widehat{{\boldsymbol{\beta }}}$, respectively. Define $\Lambda _\varepsilon (t)$, $N_{i}({\boldsymbol{\alpha }},{\boldsymbol{\beta }},t)$, $Y_{i}({\boldsymbol{\alpha }},{\boldsymbol{\beta }},t)$, $\nu _{i}({\boldsymbol{\alpha }},{\boldsymbol{\beta }},t)$, and $\widehat{\Lambda }_\varepsilon ({\boldsymbol{\alpha }},{\boldsymbol{\beta }},t)$ similar to those for the association tests, and define $M_{i,\mbox{int}}(\Lambda _\varepsilon ,{\boldsymbol{\alpha }},{\boldsymbol{\beta }})=N_{i}({\boldsymbol{\alpha }},{\boldsymbol{\beta }},\infty )-\int _{-\infty }^\infty \nu _{i}({\boldsymbol{\alpha }},{\boldsymbol{\beta }},t)Y_{i}({\boldsymbol{\alpha }},{\boldsymbol{\beta }},t)d\Lambda _\varepsilon (t)$ and $\widetilde{M}_{i,\mbox{int}}=M_{i,\mbox{int}}(\widehat{\Lambda }_\varepsilon (\widehat{{\boldsymbol{\alpha }}},\widehat{{\boldsymbol{\beta }}},\cdot ),\widehat{{\boldsymbol{\alpha }}},\widehat{{\boldsymbol{\beta }}})$. Following the idea in developing the association tests, the interaction tests are based on the test statistic,


(9) \begin{align*}& R_{\mbox{int}}=\widetilde{{\mathbf M}}_{\mbox{int}}^{T}({\mathbf K}\cdot{\mathbf H})\widetilde{{\mathbf M}}_{\mbox{int}},\end{align*}

where $\widetilde{{\mathbf M}}_{\mbox{int}}=(\widetilde{M}_{1,\mbox{int}},\ldots ,\widetilde{M}_{n,\mbox{int}})^{T}$, ${\mathbf K}=\{K({\mathbf G}_{i},{\mathbf G}_{j})\}_{n\times n}$, ${\mathbf H}=\{H({\mathbf X}_{i},{\mathbf X}_{j})\}_{n\times n}$, and $\cdot $ represents the Hadamard product. Here, $K({\mathbf G}_{i},{\mathbf G}_{j})={\mathbf G}_{i}^{T}{\mathbf G}_{j}$ if ${\mathbf G}$ is expected to have a linear effect under the null, and $K({\mathbf G}_{i},{\mathbf G}_{j})=\sum _{k=1}^{p}I(G_{i,k}=G_{j,k})$ if ${\mathbf G}$ is a vector of SNPs and the mode of inheritance for each SNP in ${\mathbf G}$ is unspecified. We choose $H({\mathbf X}_{i},{\mathbf X}_{j})={\mathbf X}_{i}^{T}{\mathbf X}_{j}$ if ${\mathbf X}$ is expected to have a linear effect under the null, and when ${\mathbf X}$ codes explicit subpopulations, we can also choose $H({\mathbf X}_{i},{\mathbf X}_{j})$ to be the identity kernel, i.e. $H({\mathbf X}_{i},{\mathbf X}_{j})=I({\mathbf X}_{i}={\mathbf X}_{j})$. The choices of ${\mathbf K}$ and ${\mathbf H}$ are to match the functional forms of ${\mathbf G}$ and ${\mathbf X}$ in the null model, respectively. When ${\mathbf G}$ is an SNP vector and the linear kernel is used, we center ${\mathbf G}_{i}$’s so that $\sum _{i=1}^{n}{\mathbf G}_{ij}=0$  $(j=1,\ldots ,p)$ before fitting the null model and computing $R_{int}$. This is to avoid too many zeros in the kernel matrix product ${\mathbf K}\cdot{\mathbf H}$.

The approximate null distribution of $R_{\mbox{int}}$ can be derived the same way as for $R$, except that ${\mathbf K}$ is replaced by ${\mathbf K}\cdot{\mathbf H}$ in the derivation. Based on this distribution, we compute the p-value, $P(R_{\mbox{int}}\ge R_{\mbox{int}}^{\mbox{obs}})$, using Davies’ method [17]. A gene–gene interaction test can also be developed by replacing ${\mathbf X}$ with a vector of genetic markers.

### Small-sample corrections to the proposed tests

When the sample size is limited and the null models of the proposed association and interaction tests have many adjustment covariates, which often occurs in the interaction tests as a set of genetic covariates are adjusted in the null model, the asymptotic null distributions of the test statistics may not be accurate enough to approximate their finite-sample null distributions. The proposed tests tend to be conservative or liberal depending on whether they are association tests or interaction tests, as shown in the simulations. To address this issue, we develop a small-sample correction strategy for the proposed tests, which was motivated by a similar correction strategy proposed for the kernel association tests (KAT) for quantitative traits [19]. The KAT statistic for quantitative traits takes the form $\hat{{\mathbf u}}^{T}{\mathbf K} \hat{{\mathbf u}}$, where $\hat{{\mathbf u}}$ is the residual vector of the null model. The small-sample KAT statistic in Chen *et al.* [19] is $\hat{{\mathbf u}}^{T}{\mathbf K} \hat{{\mathbf u}}/\hat{{\mathbf u}}^{T}\hat{{\mathbf u}}$. Drawing upon the similarity between our proposed test statistics and the KAT, we change the test statistics $R$, $R_{\mbox{het}}$ and $R_{\mbox{int}}$ into $R^{c}\equiv \widetilde{{\mathbf M}}^{T}{\mathbf K}\widetilde{{\mathbf M}}/{\widetilde{{\mathbf M}}^{T}\widetilde{{\mathbf M}}}$, $R_{\mbox{het}}^{c}\equiv \widetilde{{\mathbf M}}^{T}{\mathbf W}\widetilde{{\mathbf M}}/{\widetilde{{\mathbf M}}^{T}\widetilde{{\mathbf M}}}$, and $R_{\mbox{int}}^{c}\equiv \widetilde{{\mathbf M}}_{\mbox{int}}^{T}({\mathbf K}\cdot{\mathbf H})\widetilde{{\mathbf M}}_{\mbox{int}}/{\widetilde{{\mathbf M}}_{\mbox{int}}^{T}\widetilde{{\mathbf M}}_{\mbox{int}}}$, respectively for small-sample correction. The p-value, $P(R^{c}\ge R^{c}_{\mbox{obs}})$, equals $P(\widetilde{{\mathbf M}}^{T}({\mathbf K}-R^{c}_{\mbox{obs}}{\mathbf I}_{n})\widetilde{{\mathbf M}}\ge 0)$, where ${\mathbf I}_{n}$ is an $n\times n$ identity matrix. The large-sample null distribution of $\widetilde{{\mathbf M}}^{T}({\mathbf K}-R^{c}_{\mbox{obs}}{\mathbf I}_{n})\widetilde{{\mathbf M}}$ can be obtained following the derivation for $R$, which is also a linear combination of independent chi-square variables with degree 1. So we use Davies’ method [17] to compute the p-value. The p-values, $P(R_{\mbox{het}}^{c}\ge R^{c}_{\mbox{het,obs}})$ and $P(R_{\mbox{int}}^{c}\ge R^{c}_{\mbox{int,obs}})$, can be computed similarly.

## Numerical experiments

We performed Monte Carlo simulations to assess the finite-sample performance of the proposed association tests with competing risk data under left truncation. In all the simulation scenarios, two competing risks were considered. The competing risk data were generated from the AFT models specified later in specific scenarios, following the steps in Section 3.2 of Beyersmann *et al.* [20]. The left-truncation time was generated from $U(0,1)$, and the residual censoring time (censoring time since truncation) was generated from $\exp (0.1)$. The failure due to Cause 1 is of interest. When considering adjustment covariates, a binary covariate $Z_{1} \sim Bernoulli(0.5)$ and a continuous covariate $Z_{2} \sim U(0,2)$ were generated for each subject. The genetic markers under testing were SNPs, except in the scenario of confounding where the genetic markers were gene expression values. We generate SNP covariates by sampling from the genotype data of the 1000 genomes project (phase 3) [21]. In all the simulation scenarios, 1000 Monte Carlo samples were generated, and the significance level of a test was set at 0.05, unless otherwise specified.

In Supplementary Appendix B, we provided additional simulations regarding the interaction tests, the small-sample adjustment, the empirical sizes of the association tests under stringent p-value thresholds, the association test considering genetic heterogeneity when the subpopulations are latent, the Cox model-based kernel association test (coxKM) [6] with data generated from an AFT model having four adjustment covariates, and the runtimes of the interaction tests and the small-sample adjusted versions of all the proposed tests.

### Testing genetic association in the absence of genetic heterogeneity

In this series of simulations, the performance of the test $R$ was assessed in detecting the association between a set of genetic markers and the failure due to Cause 1 in the absence of genetic heterogeneity. The hazard function of Cause 1 followed an AFT model,


(10) \begin{align*} \lambda_{1} (t|{\mathbf Z},{\mathbf G}) =& \lambda_{0}\left(t\cdot \exp\left \{-\sum_{j=1}^{p} \beta_{j} G_{j} - \sum_{k=1}^{2} 0.1 Z_{k}\right\}\right) \nonumber \\ &\exp\left \{-\sum_{j=1}^{p} \beta_{j} G_{j} - \sum_{k=1}^{2} 0.1 Z_{k}\right\},\end{align*}

and the hazard of Cause 2 followed another AFT model,


(11) \begin{align*} \lambda_{2} (t|{\mathbf Z},{\mathbf G}) =& \lambda_{0}\left(t \cdot \exp\left \{-\sum_{j=1}^{p} \alpha_{j} G_{j} - \sum_{k=1}^{2} 0.2 Z_{k}\right\}\right) \nonumber \\ &\exp\left \{-\sum_{j=1}^{p} \alpha_{j} G_{j} - \sum_{k=1}^{2} 0.2 Z_{k}\right\},\end{align*}

where the values of $\beta _{j}$’s and $\alpha _{j}$’s varied depending on the simulation scenario. The baseline hazard function was $\lambda _{0}(x) = x^{2} + x$.

#### Empirical size and power of the test $R$ under no confounding

In this subset of simulations, we investigated the performance of the test $R$ in the absence of genetic heterogeneity and confounding effects under various $n$’s and $p$’s. For comparison, we also investigated the performance of $R_{\mbox{het}}$ in the same settings as for $R$. The genetic markers under testing were SNPs. We set the regression coefficients of ${\mathbf G}$ in (1) to be $\beta _{j}=0.08$ and $\beta _{j}=0$ ($j=1,\ldots ,p$) in the power evaluation and the size assessment, respectively, and set the regression coefficients of ${\mathbf G}$ in (2) to be $\alpha _{j}=0.16$ ($j=1,\ldots ,p$). The IBS kernel was used to measure the genetic similarity in $R$ and $R_{\mbox{het}}$. The Gaussian kernel was used to measure the subpopulation similarity in $R_{\mbox{het}}$, where the population structure was represented by the adjustment covariates.

Table 1 shows that the empirical sizes of both $R$ and $R_{\mbox{het}}$ are close to the nominal level under various $n$’s and $p$’s. The powers of $R$ and $R_{\mbox{het}}$ both increase with the sample size, and the former is a little higher than the latter due to the unnecessary accounting for heterogeneity by $R_{\mbox{het}}$ in this scenario. To investigate how the dimension of markers affect the power of the proposed association tests, we conducted a simulation study same as for Table 1 except that the SNP sets were expanded with 15 no-effect (noise) SNPs (to fix the censoring rate). As shown in Table 2, the tests $R$ and $R_{\mbox{het}}$ still control Type I error well, but they have lower power than that in Table 1. Similar observations were made in kernel-based two-sample and independence testing in high dimensions [22].

**Table 1:** Empirical size and power comparison of $R$ and $R_{\mbox{het}}$ in testing genetic effects under covariate adjustment, left truncation and no genetic heterogeneity

	Empirical size (Power)	
	$\boldsymbol{p=20}$ , $\boldsymbol{n=400}$	$\boldsymbol{p=20}$ , $\boldsymbol{n=500}$
$R$	0.044 (0.447)	0.052 (0.574)
$R_{\mathrm{het}}$	0.045 (0.426)	0.049 (0.537)
	$\boldsymbol{p=25}$ , $\boldsymbol{n=400}$	$\boldsymbol{p=25}$ , $\boldsymbol{n=500}$
$R$	0.046 (0.540)	0.042 (0.646)
$R_{\mathrm{het}}$	0.042 (0.476)	0.046 (0.603)

**Table 2:** Empirical size and power comparison of $R$ and $R_{\mbox{het}}$ in testing genetic effects under covariate adjustment, left truncation and no genetic heterogeneity. In each scenario, there are 15 noise SNPs

	Empirical size (Power)	
	$\boldsymbol{p=35}$ , $\boldsymbol{n=400}$	$\boldsymbol{p=35}$ , $\boldsymbol{n=500}$
$R$	0.046 (0.345)	0.036 (0.445)
$R_{\mathrm{het}}$	0.044 (0.298)	0.034 (0.389)
	$\boldsymbol{p=40}$ , $\boldsymbol{n=400}$	$\boldsymbol{p=40}$ , $\boldsymbol{n=500}$
$R$	0.046 (0.439)	0.037 (0.544)
$R_{\mathrm{het}}$	0.035 (0.372)	0.035 (0.489)

We also compared $R$ and $R_{\mbox{het}}$ with six existing multi-marker survival tests in terms of empirical size, power, and runtime, which are coxKM [6], coxSKATs [7], Global Test [5], Wald [14], $WV_{\mathrm{coxph}}$[8], and aftKM [9]. Because aftKM does not handle left-truncated survival data, and the code from Sinnott and Cai [9] lacks the option to use IBS kernel for genetic similarity, the comparisons were divided into two simulation scenarios. Table 3 shows the comparison of all the tests excluding aftKM under left truncation, where the IBS genetic similarity kernel was used in all the considered tests except Global Test and Wald. Table 4 shows the comparison of all the tests under no left truncation, where Gaussian genetic similarity kernel was used in all the considered tests except Global Test and Wald. The runtime comparison was conducted under the latter scenario, and the results are shown in Table 5. Note that Global Test [5] assumes a linear genetic effect and uses the linear kernel for genetic similarity. As requested by a referee, here we added a setting of $p=35$ and $n=1500$ to assess the performance of the proposed association tests with larger-scale data. We chose $p=35$ because it is the 90th percentile of the SNP set size in the ROSMAP data and $n=1500$ because it is close to the size of the analytic sample of ROSMAP, which is 1440. All the tests were executed on a workstation with a 28-core CPU at 2.40 GHz and 115 GB RAM. The programming language was R, but we used the R package Rcpp, which offers a seamless integration of R and C++, to reduce the runtime.

**Table 3:** Empirical size and power comparison of $R$, $R_{\mbox{het}}$, coxKM, coxSKATs, Global Test, Wald, and $WV_{\mathrm{coxph}}$ in testing genetic effects under covariate adjustment, left truncation, and no genetic heterogeneity

	Empirical size (Power)				
	$\boldsymbol{p=20}$ , $\boldsymbol{n=400}$	$\boldsymbol{p=25}$ , $\boldsymbol{n=400}$	$\boldsymbol{p=20}$ , $\boldsymbol{n=500}$	$\boldsymbol{p=25}$ , $\boldsymbol{n=500}$	$\boldsymbol{p=35}$ , $\boldsymbol{n=1500}$
$R$	0.044 (0.447)	0.046 (0.540)	0.052 (0.574)	0.042 (0.646)	0.042 (0.999)
$R_{\mathrm{het}}$	0.045 (0.426)	0.042 (0.476)	0.049 (0.537)	0.046 (0.603)	0.050 (0.999)
coxKM	0.035 (0.428)	0.028 (0.477)	0.036 (0.547)	0.022 (0.630)	0.038 (1.000)
coxSKATs	0.033 (0.406)	0.031 (0.451)	0.031 (0.529)	0.023 (0.604)	0.041 (1.000)
Global Test	0.025 (0.478)	0.030 (0.549)	0.022 (0.573)	0.022 (0.686)	0.028 (1.000)
Wald	0.073 (0.424)	0.101 (0.554)	0.083 (0.524)	0.104 (0.681)	0.208 (1.000)
$WV_{\mathrm{coxph}}$	0.050 (0.503)	0.051 (0.564)	0.041 (0.588)	0.040 (0.696)	0.054 (1.000)

**Table 4:** Empirical size and power comparison of $R$, $R_{\mbox{het}}$, coxKM, coxSKATs, Global Test, Wald, $WV_{\mathrm{coxph}}$, and aftKM in testing genetic effects under covariate adjustment, no left truncation, and no genetic heterogeneity

	Empirical size (Power)				
	$\boldsymbol{p=20}$ , $\boldsymbol{n=400}$	$\boldsymbol{p=25}$ , $\boldsymbol{n=400}$	$\boldsymbol{p=20}$ , $\boldsymbol{n=500}$	$\boldsymbol{p=25}$ , $\boldsymbol{n=500}$	$\boldsymbol{p=35}$ , $\boldsymbol{n=1500}$
$R$	0.048 (0.229)	0.042 (0.248)	0.050 (0.330)	0.047 (0.338)	0.042 (0.915)
$R_{\mathrm{het}}$	0.043 (0.209)	0.043 (0.220)	0.049 (0.301)	0.046 (0.317)	0.047 (0.910)
coxKM	0.023 (0.214)	0.029 (0.217)	0.024 (0.302)	0.023 (0.303)	0.028 (0.922)
coxSKATs	0.024 (0.210)	0.036 (0.239)	0.041 (0.303)	0.040 (0.329)	0.042 (0.916)
Global Test	0.032 (0.237)	0.042 (0.281)	0.045 (0.310)	0.047 (0.352)	0.037 (0.930)
Wald	0.155 (0.404)	0.220 (0.503)	0.147 (0.435)	0.195 (0.553)	0.265 (0.980)
$WV_{\mathrm{coxph}}$	0.034 (0.268)	0.044 (0.306)	0.049 (0.344)	0.048 (0.382)	0.040 (0.937)
aftKM	0.016 (0.144)	0.015 (0.152)	0.028 (0.210)	0.018 (0.205)	0.021 (0.820)

**Table 5:** Runtime comparison of $R$, $R_{\mbox{het}}$, coxKM, coxSKATs, Global Test, Wald, $WV_{\mathrm{coxph}}$, and aftKM in testing genetic effects under covariate adjustment, no left truncation and no genetic heterogeneity

	Runtime (seconds)				
	$\boldsymbol{p=20}$ , $\boldsymbol{n=400}$	$\boldsymbol{p=25}$ , $\boldsymbol{n=400}$	$\boldsymbol{p=20}$ , $\boldsymbol{n=500}$	$\boldsymbol{p=25}$ , $\boldsymbol{n=500}$	$\boldsymbol{p=35}$ , $\boldsymbol{n=1500}$
$R$	2.64	2.44	3.51	3.61	49.54
$R_{\mathrm{het}}$	2.86	2.75	3.86	3.88	43.43
coxKM	0.31	0.26	0.37	0.36	2.30
coxSKATs	0.06	0.06	0.10	0.11	1.58
Global Test	0.03	0.03	0.04	0.05	0.51
Wald	56.89	83.60	86.87	127.51	2001.96
$WV_{\mathrm{coxph}}$	0.02	0.02	0.04	0.03	0.36
aftKM	47.27	44.88	48.87	48.46	135.01

Tables 3 and 4 show that $R$ and $R_{\mbox{het}}$ perform as theoretically expected in terms of size and power across all the settings. $WV_{\mathrm{coxph}}$ performs well too, since it has certain robustness against model misspecification, as explained by Li *et al.* [8]. However, $WV_{\mathrm{coxph}}$ cannot adjust for nonlinear confounding, as discussed in the subsection ‘Association tests’. An inflated Type I error rate was observed for $WV_{\mathrm{coxph}}$ under nonlinear confounding in a simulation not shown here due to space limitations. The Wald test does not control Type I error rate under the nominal level, because the numbers of regression coefficients in the AFT models are large. aftKM is too conservative in terms of size and has remarkably lower power than $R$ and $R_{\mbox{het}}$. coxKM, coxSKATs, and Global Test have notably smaller empirical sizes than the nominal level, which could be due to them misspecifying the null model. coxKM and coxSKATs have smaller power than $R$ when the sample size is not very large, especially under left truncation. Global Test has marginally higher power than $R$ in most of the settings, probably because the linear kernel it uses matches the true genetic effect form. In another simulation presented in Section B.6 of the supplementary material, we increased the number of adjustment covariates to further check the performance of coxKM when applied to data from AFT models, and found that coxKM was very conservative in terms of both size and power. We expect coxSKATs and Global Test to be also very conservative in that scenario since they use the same/similar test statistics to coxKM. Table 5 indicates that $R$ and $R_{\mbox{het}}$ are not as fast as those Cox model-based multi-marker tests. This is mainly because they use a resampling method twice in deriving the null distributions of the test statistics, as elaborated in Supplementary Appendix A. Nevertheless, they are much faster than the AFT model-based tests, Wald, and aftKM.

#### Empirical size and power of the test $R$ under quadratic confounding

**Figure 2:**
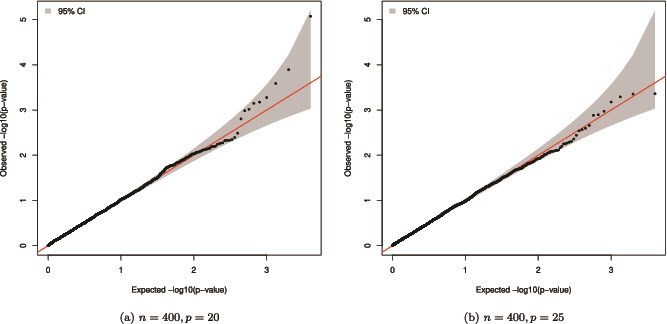
Log-scale uniform Q-Q plots of the null p-value of $R$ under quadratic confounding with $n=400$ and $p=20$ or 25.

In this simulation, the adjustment covariates $Z_{1}$ and $Z_{2}$ were confounders, and the genetic markers under testing were gene expressions. To simulate confounding effects, we assume ${\mathbf G}_{i} = \mathbf{0.5}Z_{i1} + \mathbf{0.5}Z_{i2} + \mathbf{0.25}Z_{i1}^{2} + \mathbf{0.25}Z_{i2}^{2} + \mathbf{0.5}Z_{i1}Z_{i2} + {\mathbf e}_{i}$, where ${\mathbf G}_{i}=(G_{i1},\dots ,G_{ip})^{T}$ were the expression levels of $p$ genes in subject $i$, $\mathbf{0.25}$ and $\mathbf{0.5}$ are $p$-dimensional vectors of 0.25’s and 0.5’s, respectively, and ${\mathbf e}_{i}=(e_{i1},\dots ,e_{ip})^{T}$ follows a multivariate normal distribution with a zero mean and the covariance matrix being ${\boldsymbol{\Sigma }}_{p\times p}=\{0.1^{|k-l|}\}$. The corresponding confounding effect of $Z_{1}$ and $Z_{2}$ is called quadratic confounding. As discussed in the subsection ‘Association tests’, many of the existing multi-marker survival tests, including Cai *et al.* [6] and Sinnott and Cai [9], cannot adjust for confounding at all, and the tests of Li *et al.* [8] can only adjust for linear confounding. So we use simulations under quadratic confounding to illustrate that our association test $R$ can adjust for confounding regardless of the relationship between the genetic markers and the confounders. The regression coefficients of ${\mathbf G}$ in (1) were set to be $\beta _{j}=0$ for the size assessment and $\beta _{j}=0.03$ for the power evaluation ($j=1,\ldots ,p$). In the hazard function (2), we set $\alpha _{j}=0.1$ for both the empirical size and power evaluations. We used the Gaussian kernel to measure the gene expression similarity in $R$. Figure 2 shows that the p-value of $R$ under the null follows a $U[0,1]$ distribution when adjusting for confounders. Supplementary Table 3 shows that under quadratic confounding, the empirical size of $R$ is still close to the nominal level, and the power of the test increases with the sample size.

### Testing genetic association in the presence of genetic heterogeneity

In this simulation, we investigated the empirical size and power of $R_{\mbox{het}}$ for testing the joint effect of an SNP set in the presence of genetic heterogeneity across two observable subpopulations with equal proportions. For comparison, we also investigated the performance of $R$ in the same settings. Since we generated the SNPs by sampling from the 1000 Genomes data set, we let the two observable subpopulations be males and females and associated a sampled SNP set with the subpopulation indicated by the sex of the subject from whom the SNP set was obtained.

The survival time of Subject $i$  $(i=1,\ldots ,n)$ was generated from the following AFT models for the CSH functions,


(12) \begin{align*} \lambda_{1} (t|Z_{i},{\mathbf G}_{i}) =&\ \lambda_{0}\left(t\cdot \exp\left \{-\sum_{j=1}^{p} (\beta_{0} + \beta_{1} Z_{i})G_{ij} - 0.5 Z_{i}\right\}\right) \nonumber \\ &\exp \left\{-\sum_{j=1}^{p} (\beta_{0} + \beta_{1} Z_{i})G_{ij} - 0.5 Z_{i}\right\}, \end{align*}

(13) \begin{align*} \lambda_{2} (t|Z_{i},{\mathbf G}_{i}) =&\ \lambda_{0}\left(t\cdot \exp\left \{-\sum_{j=1}^{p} 0.2 G_{ij} - Z_{i}\right\}\right) \nonumber \\ &\exp\left \{-\sum_{j=1}^{p} 0.2 G_{ij} - Z_{i}\right\}, \end{align*}

where $Z_{i}$ is a Bernoulli random variable with success probability 0.6. To assess the size and the power of $R_{\mbox{het}}$, we set $\beta _{0} = 0$ and $\beta _{0} = 0.002$, respectively. We also set $\beta _{1}=0$ in the size assessment and $\beta _{1} = 0.1$ and $ 0.2$ in the power assessment, with the larger $\beta _{1}$ representing the stronger genetic heterogeneity. The IBS kernel was used to measure the genetic similarity in $R_{\mbox{het}}$ and $R$, and the identity kernel $I(Z_{i}=Z_{j})$ was used to measure the subpopulation similarity in $R_{\mbox{het}}$.

Table 6 shows that the empirical sizes of $R_{\mbox{het}}$ and $R$ are both around the nominal level. Table 6 also shows that $R_{\mbox{het}}$ has higher power than $R$ by accounting for the genetic heterogeneity across the two observable subpopulations. As the heterogeneity size (measured by $\beta _{1}$) increases, the power advantage of $R_{\mbox{het}}$ against $R$ becomes more obvious. Readers may ask why the power changes differently in the different settings of Table 6 as the value of $p$ increases. The possible reasons are the following. While increasing the number of causal SNPs potentially leads to a power increase, it also increases the dimensionality, which potentially reduce the power for kernel-based tests [23]. Also, in the setting of $\beta _{1}=0.1$, increasing $p$ decreased the censoring rate ($P(\epsilon \Delta =0)$) by $1\%$, whereas when $\beta _{1}=0.2$, increasing $p$ increased the censoring rate by $2\%$. We also investigated the consequence of using a variable $Z\sim Bernoulli(0.5)$ independent of the one in (3) and (4) to replace the latter in $R$ and $R_{\mbox{het}}$, i.e. using a non-informative subpopulation indicator. Compared with Table 6, Table 7 suggests that this mistake does not affect the sizes of $R$ and $R_{\mbox{het}}$, but it significantly reduces the power of $R_{\mbox{het}}$, and it also reduces the power of $R$ when the heterogeneity size is not small.

**Table 6:** Empirical sizes and powers of $R_{\text{{het}}}$ and $R$ in testing genetic association under genetic heterogeneity across two observable subpopulations and left truncation

	Size/Power					
	$\boldsymbol{p=20, n=400}$			$\boldsymbol{p=20, n=500}$		
($\beta _{0}$, $\beta _{1}$)	(0, 0)	(0.002, 0.1)	(0.002, 0.2)	(0, 0)	(0.002, 0.1)	(0.002, 0.2)
$R$	0.042	${\color{blue}{0.256}}$	${\color{blue}{0.505}}$	0.052	${\color{blue}{0.344}}$	${\color{blue}{0.621}}$
$R_{\mbox{het}}$	0.042	${\color{blue}{0.326}}$	${\color{blue}{0.665}}$	0.047	${\color{blue}{0.444}}$	${\color{blue}{0.762}}$
	$\boldsymbol{p=25, n=400}$			$\boldsymbol{p=25, n=500}$		
($\beta _{0}$, $\beta _{1}$)	(0, 0)	(0.002, 0.1)	(0.002, 0.2)	(0, 0)	(0.002, 0.1)	(0.002, 0.2)
$R$	0.047	${\color{blue}{0.295}}$	${\color{blue}{0.485}}$	0.050	${\color{blue}{0.372}}$	${\color{blue}{0.575}}$
$R_{\mbox{het}}$	0.041	${\color{blue}{0.375}}$	${\color{blue}{0.618}}$	0.045	${\color{blue}{0.482}}$	${\color{blue}{0.735}}$

**Table 7:** Empirical sizes and powers of $R_{\mbox{het}}$ and $R$ in testing genetic association under genetic heterogeneity across two observable subpopulations and left truncation. The tests were performed with a Bernoulli random variable different from that employed for data generation

	Size/Power					
	$\boldsymbol{p=20, n=400}$			$\boldsymbol{p=20, n=500}$		
($\beta _{0}$, $\beta _{1}$)	(0, 0)	(0.002, 0.1)	(0.002, 0.2)	(0, 0)	(0.002, 0.1)	(0.002, 0.2)
$R$	0.046	0.264	0.396	0.044	0.352	0.492
$R_{\mbox{het}}$	0.051	0.074	0.096	0.048	0.075	0.125
	$\boldsymbol{p=25, n=400}$			$\boldsymbol{p=25, n=500}$		
($\beta _{0}$, $\beta _{1}$)	(0, 0)	(0.002, 0.1)	(0.002, 0.2)	(0, 0)	(0.002, 0.1)	(0.002, 0.2)
$R$	0.044	0.289	0.341	0.046	0.369	0.450
$R_{\mbox{het}}$	0.047	0.076	0.087	0.047	0.085	0.109

## A real application

We applied our tests $R^{c}$ and $R^{c}_{\text{{het}}}$ to the GWAS and AD diagnosis data from two large longitudinal studies of aging and dementia, the Religious Orders Study (ROS) and the Rush Memory and Aging Project (MAP) [13], collectively called ROSMAP. The goal of this real data analysis is to discover genes that are associated with age at AD onset. Death before AD onset is a competing risk. We excluded the subjects who had AD at the baseline visit from the analysis, leading to left truncation with the baseline age as the truncation time. Both the competing risk and the left truncation were accounted for in our analysis.

The GWAS dataset includes 1679 subjects and 750 173 SNPs. After performing SNP-level quality control—removing SNPs with minor allele frequency (MAF)$<0.01$, Hardy–Weinberg equilibrium test’s p-value$<10^{-6}$, or missing rate$>0.02$, 619 061 SNPs remained for the analysis. We then performed subject-level quality control to remove subjects with missing SNP genotype rate$>0.02$. After that, 1618 subjects remained for the analysis. The missing genotypes in the remaining genetic data were imputed by IMPUTE v2.3.2 (https://mathgen.stats.ox.ac.uk/impute/impute_v2.html#download) with prephasing [24]. Then the 619 061 SNPs were grouped into gene-based SNP sets based on the human genome reference hg18 (i.e. located in or within 5K base pairs upstream/downstream of a gene). The grouping formed 21 285 genes along with the *APOE* gene, which was coded as the count of *APOE*-$\epsilon 4$ alleles. Supplementary Table 17 summarizes the distribution of the SNP set size. We performed a principal component analysis of the imputed GWAS data to obtain the first six principal components for adjusting for population stratification. The AD diagnosis dataset we used from the ROS and MAP studies was frozen in 2021 with a sample size of 3675. It contains annual clinical diagnosis of AD since baseline. Out of the 3675 subjects, 218 subjects had AD at baseline and thus were removed from the analysis. We treated the age at the first diagnosis of AD as the age at AD onset. We then merged the age at AD onset data with the processed GWAS data to generate the final analysis-ready data, which contains 1440 subjects who have both the genetic data and the survival data. Among them, 540 subjects developed AD during the follow-up. In the analysis, to improve power and/or reduce confounding, we adjusted for the first six principal components from the GWAS data as well as sex, cohort (ROS or MAP), and education attainment (0: $\mbox{years of education}\le 12$; 1: $12<\mbox{years of education}\le 16$; 2: $\mbox{years of education}>16$). We did not adjust for self-reported race because all but one of the 1440 subjects reported to be white.

Without considering genetic heterogeneity, we used the test $R^{c}$ to perform a genome-wide gene-based association scan by testing the effect of each of the 21 286 genes on age at AD onset. Four genetic similarity kernels were used: the IBS, linear, Laplacian, and quadratic kernels. Controlling the false discover rate under 10% by the Benjamini–Yekutieli procedure [25], *APOE4* and *APOC1* appeared to be two significant genes no matter which genetic similarity kernel was used (Table 8). *APOE4*’s $p$-value ranged from 1.11E-16 to 1.34E-13, and *APOC1*’s $p$-value ranged from 2.03E-10 to 5.01E-09. *APOE4* is an established susceptibility gene for AD, and *APOC1* has also been reported to be an AD risk variant (see, e.g. Kulminski *et al.*[26] and Zhou *et al.*[27]).

**Table 8:** Top five genes discovered by $R^{c}$ and $R^{c}_{\mbox{het}}$ with the ROSMAP data. IBS, Lin, Lap, and Quad stand for the IBS, linear, Laplacian, and quadratic kernels, respectively. Various types of heterogeneity were considered, including no genetic heterogeneity (S1), heterogeneity between sexes (S2), heterogeneity across education attainment categories (S3), and heterogeneity across genetic backgrounds (S4)

Genetic similarity kernel	Scenario			Genes and *p*-values		
	S1	**APOE4**	**APOC1**	IGSF23	PLEKHG5,TNFRSF25	TBCC
		**2.41E-14**	**4.61E-09**	4.99E-05	7.91E-05	8.11E-05
IBS	S2	**APOE4**	**APOC1**	PLEKHG5,TNFRSF25	IGSF23	TBCC
		**8.04E-14**	**3.25E-09**	5.64E-05	1.35E-04	1.42E-04
	S3	**APOE4**	**APOC1**	IGSF23	TBCC	PLEKHG5
						TNFRSF25
		**3.01E-14**	**2.53E-09**	4.50E-05	4.84E-05	7.17E-05
	S4	**APOE4**	**APOC1**	IGSF23	PLEKHG5,TNFRSF25	TBCC
		**2.38E-14**	**1.59E-09**	5.01E-05	7.94E-05	8.11E-05
	S1	**APOE4**	**APOC1**	IGSF23	MTMR2	HSBP1
		**1.11E-16**	**2.03E-10**	3.12E-05	9.79E-05	1.15E-04
Lin	S2	**APOE4**	**APOC1**	IGSF23	MTMR2	GRIP1
		**2.00E-15**	**8.31E-10**	6.73E-05	1.43E-04	1.52E-04
	S3	**APOE4**	**APOC1**	IGSF23	HSBP1	MTMR2
		**6.33E-15**	**4.78E-10**	3.30E-05	1.04E-04	1.22E-04
	S4	**APOE4**	**APOC1**	IGSF23	MTMR2	HSBP1
		**4.44E-16**	**1.99E-10**	3.18E-05	9.92E-05	1.13E-04
	S1	**APOE4**	**APOC1**	IGSF23	GRIP1	TBCC
		**1.34E-13**	**5.01E-09**	4.56E-05	1.14E-04	1.53E-04
Lap	S2	**APOE4**	**APOC1**	IGSF23	PLEKHG5,TNFRSF25	GRIP1
		**2.59E-13**	**1.23E-08**	1.31E-04	1.40E-04	1.65E-04
	S3	**APOE4**	**APOC1**	IGSF23	TBCC	GRIP1
		**2.03E-13**	**4.80E-09**	3.88E-05	9.40E-05	1.20E-04
	S4	**APOE4**	**APOC1**	IGSF23	GRIP1	TBCC
		**1.37E-13**	**5.95E-09**	4.58E-05	1.15E-04	1.53E-04
	S1	**APOE4**	**APOC1**	EHHADH-AS1	GRIP1	GABBR1
		**1.33E-15**	**2.99E-10**	1.18E-04	1.48E-04	1.72E-04
Quad	S2	**APOE4**	**APOC1**	EHHADH-AS1	GRIP1	C16orf54
		**1.11E-15**	**1.67E-09**	7.80E-05	1.32E-04	1.96E-04
	S3	**APOE4**	**APOC1**	EHHADH-AS1	GABBR1	GRIP1
		**4.44E-16**	**3.55E-10**	1.38E-04	1.44E-04	1.45E-04
	S4	**APOE4**	**APOC1**	EHHADH-AS1	GRIP1	GABBR1
		**4.44E-16**	**2.23E-10**	1.16E-04	1.48E-04	1.69E-04
p-value threshold^a^		4.46E-07	8.91E-07	1.34E-06	1.78E-06	2.23E-06

$^{\mathrm{a}}$ FDR-based p-value thresholds were calculated according to Benjamini and Yekutieli [25] under arbitrary dependence assumption, i.e. $\mathrm{Threshold}_{i} = \frac{\alpha i}{m\sum _{k=1}^{m} (1/k)}\ (i=1,\ldots ,m)$, where $m$ is the number of tests and $\alpha $ is the target FDR.

We further performed genome-wide gene-based association analyses by using $R^{c}_{\mbox{het}}$ to consider three types of genetic heterogeneity, namely genetic heterogeneity due to different sexes, education levels, and genetic backgrounds (S2–S4 in Table 8). When considering heterogeneity across different genetic backgrounds, we randomly selected 200 000 SNPs from the whole genome to measure the genetic background. *APOE* and *APOC1* remained to be the only two genome-wide significant genes under the consideration of each type of genetic heterogeneity (Table 8).

Although not reaching the genome-wide significance level, *IGSF23* was most frequently found to be the third ranking genei.e. associated with age at AD onset in our analyses (Table 8). It was also implicated in a recent study of AD genetics [28].

The finding that *APOE* and *APOC1* are the only genome-wide significant genes with and without considering multiple heterogeneity sources could be due to three reasons. First, the sample size of the ROSMAP analysis ($n=1440$) is relatively modest for a GWAS. While *APOE* has a large effect on AD risk, which may be detectable in smaller samples, other AD-associated genes typically have much weaker effects that require larger cohorts to satisfy the stringent genome-wide significance threshold. The fact that *IGSF23*, a gene implicated in a recent GWAS [28], was consistently ranked among the top genes by our methods suggests that the tests are prioritizing biologically relevant candidates, even if they fail to reach the genome-wide significance level due to power limitations. Second, there may be genetic heterogeneity, but the specified subpopulation indicators (sex, education, and genetic background) do not accurately reflect the underlying heterogeneity. As shown in our simulation, using a non-informative subpopulation indicator would substantially reduce the power of $R_{\mbox{het}}$, and likely $R^{c}_{\mbox{het}}$ as well. Third, the proportion of non-causal SNPs in a gene may be considerable. Our simulation has indicated that the powers of $R$ and $R_{\mbox{het}}$ are attenuated in the presence of no-effect SNPs; the tests $R^{c}$ and $R^{c}_{\mbox{het}}$ may be similarly affected.

Genomic p-value Q-Q plots from Supplementary Figures 7–11 and the estimated genomic inflation factors in Supplementary Table 18 suggest that there is no population stratification or cryptic relatedness affecting the association testing results of $R^{c}$ and $R^{c}_{\mbox{het}}$. These figures and the table also indicate that $R^{c}$ and $R^{c}_{\mbox{het}}$ have no other systematic bias when applied to the ROSMAP data, except that $R^{c}_{\mbox{het}}$ is conservative when considering heterogeneity between sexes with IBS or Laplacian kernel for genetic similarity in the ROSMAP analysis. The cause for this conservative shift has not yet been identified.

Finally, we used $R^{c}_{\mbox{int}}$ to test for the pair-wise interaction between the top-three genes frequently identified by our association tests, *APOE4*, *APOC1,* and *IGSF23*, and for their interactions with sex and education attainment, respectively. The $p$-values of those interaction tests are shown in Supplementary Table 19. None of the interactions is statistically significant at the 0.05 level.

## Discussion

We have developed a suite of novel genetic association and interaction tests for survival outcomes based on the AFT model. When the data follow an AFT model, the proposed tests have correct Type I error rates under finite samples of realistic sizes and are able to handle complex confounding, competing risks, and left truncation. Furthermore, the new association tests can account for genetic heterogeneity to improve the power of association discovery. However, we caution readers that the performance of the proposed tests may deteriorate when the data violate the AFT assumption.

Based on our comparisons of the proposed association tests and the existing multi-marker survival tests, we suggest using the tests $R$ and $R_{\mbox{het}}$ rather than coxKM, coxSKATs, Global Test, and $WV_{\mathrm{coxph}}$ in set-based genome-wide association testings when the phenotype variable is expected to follow an AFT model rather than a Cox model—as using those Cox-based tests may lead to reduced power (coxKM, coxSKATs, and Global Test) or inflated Type I error rates ($WV_{\mathrm{coxph}}$). The choice between $R$ and $R_{\mbox{het}}$ depends on whether there is genetic heterogeneity and whether the subpopulation can be correctly represented by observable variables. When the underlying survival model is uncertain but there is no confounding or only linear confounding, one might employ $WV_{\mathrm{coxph}}$ as it is robust against model misspecification to a certain extent. The Wald test for the AFT model should only be adopted for multi-marker association testing when the marker set size is small, say, less than five, since it would otherwise have an inflated Type I error rate. Based on our simulations, those in Sinnott and Cai [9] and the discussion about confounding at the end of the subsection ‘Association tests’, we suggest using the test aftKM only when there is no confounding and the genetic markers are continuous, e.g. gene expression measurements. According to our small-sample adjustment simulations, we recommend applying the adjustment to enhance the power of association testing, especially when the sample size is small, say, <1000, and we also recommend performing the adjustment to the proposed interaction tests when the sample size is limited, say, <1500, in which scenario the unadjusted interaction tests may have inflated Type I error rates. In large samples, say $n>1000$, the unadjusted association tests may be chosen to save computation time while maintaining statistical power and accurate Type I error control compared with the adjusted versions.

Many works have demonstrated that different kernel functions could result in significantly different powers for kernel-based association tests [e.g. 16, 29, 30]. The kernel that characterizes the true functional form of genetic effect is expected to achieve the optimal power. If no information on the underlying genetic function is available, we can create a composite kernel from a set of candidate ones using the procedure in Wu *et al.* [29] or combine p-values from different kernels by the perturbation method of Wu *et al.* [29] or the ACAT method of Liu *et al.* [31] to ensure robust power.

Several future research directions related to this work are worth pursuing. First, our G-G (G-E) interaction test assumes a parametric form of the main effects of the two marker sets (the marker set and the environment variable(s)) to adapt the derivation of the asymptotic null distributions for the association tests to the interaction test. This assumption might not be true in practice. It is appealing to use the kernel trick to nonparametrically model the main effects in testing the interaction. Second, genes in a biological pathway are connected due to regulatory interactions. By leveraging this network (graph) information, which can be obtained from pathway databases like KEGG [32], we can increase the power of our tests for association and interaction involving gene sets. Third, our tests were developed for data from unrelated subjects. It is worthwhile to extend them to related individuals, which inevitably exist in family studies and biobank data. Such an extension might be accomplished by incorporating a frailty term that captures relatedness into the AFT kernel machine regression [9, 33] and borrowing the idea of Zhang and Lin [34] for marker-set testing. Fourth, when a genetic association is discovered by the test $R$ or $R_{\mbox{het}}$, one may wonder how much of the association is attributable to a specific heterogeneity source such as gene–sex interaction. A possible approach to quantify the contribution of the heterogeneity to the association is to compute the fraction of $U_{\mbox{het}}\equiv \sum _{i<j}(1+H({\mathbf X}_{i},{\mathbf X}_{j}))K({\mathbf G}_{i},{\mathbf G}_{j})\widetilde{M}_{i}\widetilde{M}_{j}$ coming from $U_{\mbox{int}}\equiv \sum _{i<j}H({\mathbf X}_{i},{\mathbf X}_{j})K({\mathbf G}_{i},{\mathbf G}_{j})\widetilde{M}_{i}\widetilde{M}_{j}$, i.e. the ratio $\max (0,U_{\mbox{int}})/U_{\mbox{het}}$. Here the quantity $U_{\mbox{het}}$ can be viewed as a measure of association strength considering the heterogeneity due to ${\mathbf X}$, and the quantity $U_{\mbox{int}}$ can be viewed as a strength measure of that heterogeneity. Note that when $U_{\mbox{int}}\le 0$ or the test $R_{\mbox{het}}$ with $H({\mathbf X}_{i},{\mathbf X}_{j})$ as in $U_{\mbox{het}}$ fails to reject the null, it is appropriate to say that the specific heterogeneity source does not contribute to the association. That is why we use $\max (0,U_{\mbox{int}})$ instead of $U_{\mbox{int}}$ as the numerator of the above ratio. The validity of this measure of heterogeneity contribution to association needs further investigation.

**Key Points**

- We developed kernel-based multi-marker association and interaction tests with survival phenotypes under accelerated failure time models, which have explicit asymptotic null distributions.
- We developed a small-sample correction to the proposed multi-marker survival tests, improving their accuracy when the number of markers is relatively large in consideration of the sample size.
- The proposed tests can account for left truncation and competing risks.
- The proposed association tests can consider genetic heterogeneity to improve power for association detection.

## Supplementary Material

suppl_material_bbag155

## Data Availability

The genotype data that support the findings of this study are available from the National Institute on Aging Genetics of Alzheimer’s Disease Data Storage Site. Restrictions apply to the availability of these data, which were used under license in this paper. Data are available from https://www.niagads.org/datasets/ng00029 with the permission of the National Institute on Aging Genetics of Alzheimer’s Disease Data Storage Site. The AD diagnosis data that support the findings in this paper are available from Rush Alzheimer’s Disease Center. Restrictions apply to the availability of these data, which were used under license in this paper. Data can be requested at www.radc.rush.edu. The R code implementing the proposed methods in this article is available at https://github.com/didiwu345/Multi_Marker_AFT/.
