# Supplementary material for: Multi-marker testing based on accelerated failure time models under possible left truncation and competing risks
Source: Brief Bioinform. 2026 Apr 8;27(2):bbag155. doi: 10.1093/bib/bbag155 (PMC13069905; doi:10.1093/bib/bbag155)
Supplement: suppl_material_bbag155 [file suppl_material_bbag155.pdf]

Supplementary Material for

“Multi-Marker Testing Based on Accelerated Failure Time Models Under Possible Left Truncation and Competing Risks” by Chenxi Li, Di Wu and Qing Lu

A Derivation of the Large-Sample Null Distribution of R

Write $\mathbf{E}_1$ as $\mathbf{E}_1 = (\mathbf{E}_{11}, \ldots, \mathbf{E}_{1n})$. Then

$$\begin{aligned}
\mathbf{E}_1\widetilde{\mathbf{M}} &= \sum_{i=1}^{n} \mathbf{E}_{1i}\widetilde{M}_i \\
&= \sum_{i=1}^{n} \mathbf{E}_{1i} \int_{-\infty}^{\infty} \left\{ dN_i(\widehat{\boldsymbol\beta}, t) - \frac{\nu_i(\widehat{\boldsymbol\beta}, t) Y_i(\widehat{\boldsymbol\beta}, t)}{\sum_{j=1}^{n} \nu_j(\widehat{\boldsymbol\beta}, t) Y_j(\widehat{\boldsymbol\beta}, t)} \sum_{j=1}^{n} dN_j(\widehat{\boldsymbol\beta}, t) \right\} \\
&= \sum_{i=1}^{n} \int_{-\infty}^{\infty} \left\{ \mathbf{E}_{1i} - \frac{\sum_{j=1}^{n} \mathbf{E}_{1j}\nu_j(\widehat{\boldsymbol\beta}, t) Y_j(\widehat{\boldsymbol\beta}, t)}{\sum_{j=1}^{n} \nu_j(\widehat{\boldsymbol\beta}, t) Y_j(\widehat{\boldsymbol\beta}, t)} \right\} dN_i(\widehat{\boldsymbol\beta}, t) \\
&\equiv \mathbf{Q}_n(\widehat{\boldsymbol\beta}). \qquad (1)
\end{aligned}$$

Under the null, $\widehat{\boldsymbol\beta}$ is $n^{1/2}$-consistent for $\boldsymbol\beta$ (Lai & Ying, 1991). Using similar arguments to the proof of Theorem 1(ii) in Lai and Ying (1991), it can be shown that

$$n^{-1/2}\mathbf{E}_1(\widetilde{\mathbf{M}} - \widehat{\mathbf{M}}) = n^{-1/2}\{\mathbf{Q}_n(\widehat{\boldsymbol\beta}) - \mathbf{Q}_n(\boldsymbol\beta)\} = \mathbf{B}n^{1/2}(\widehat{\boldsymbol\beta} - \boldsymbol\beta) + o_p(1 + n^{1/2}\|\widehat{\boldsymbol\beta} - \boldsymbol\beta\|), \quad (2)$$

where $\mathbf{B}$ is the asymptotic slope matrix of $n^{-1}\mathbf{Q}_n(\boldsymbol\beta)$. Following Zeng and Lin (2008), we use a least squares method to estimate $\mathbf{B}$. Specifically, let $\widetilde{\boldsymbol\beta} = \widehat{\beta} + n^{-1/2}\mathbf{W}$, where $\mathbf{W} \sim N(\mathbf{0}, \mathbf{I}_q)$ and $\mathbf{I}_q$ is a $q \times q$ identity matrix. Equation (2) implies that

$$n^{-1/2}\{\mathbf{Q}_n(\widetilde{\boldsymbol\beta}) - \mathbf{Q}_n(\widehat{\boldsymbol\beta})\} = \mathbf{B}n^{1/2}(\widetilde{\boldsymbol\beta} - \widehat{\boldsymbol\beta}) + o_p(1) = \mathbf{B}\mathbf{W} + o_p(1). \quad (3)$$

The least squares method has the following steps:

Step 1: Generate L, say 10,000, independent realizations of $\mathbf{W}$, denoted by $\mathbf{W}_1, \ldots, \mathbf{W}_L$.

Step 2: Calculate $n^{-1/2}\{\mathbf{Q}_n(\widehat{\boldsymbol\beta} + n^{-1/2}\mathbf{W}_l) - \mathbf{Q}_n(\widehat{\boldsymbol\beta})\}$ $(l = 1, \ldots, L)$.

Step 3: For $j = 1, \ldots, m$, regress $n^{-1/2}\{\mathbf{Q}_n(\widehat{\boldsymbol\beta} + n^{-1/2}\mathbf{W}_l) - \mathbf{Q}_n(\widehat{\boldsymbol\beta})\}_j$ onto $\mathbf{W}_l$ $(l = 1, \ldots, L)$ to estimate $\mathbf{B}_j$, the j-th row of $\mathbf{B}$, using the least squares estimation.

Denote the estimator of $\mathbf{B}$ by $\widehat{\mathbf{B}}$. Recall that $\widehat{\boldsymbol{\beta}}$ is obtained from Eq. (5) in Chiou and Xu (2017) with log-rank weights,

$$\mathbf{U}_n(\boldsymbol{\beta}) \equiv \frac{1}{n}\sum_{i=1}^{n}\int_{-\infty}^{\infty}\left\{\mathbf{Z}_i - \frac{\sum_{j=1}^{n}\mathbf{Z}_j\nu_j(\boldsymbol{\beta},t)Y_j(\boldsymbol{\beta},t)}{\sum_{j=1}^{n}\nu_j(\boldsymbol{\beta},t)Y_j(\boldsymbol{\beta},t)}\right\}dN_i(\boldsymbol{\beta},t) = \mathbf{0}. \tag{4}$$

Chiou and Xu (2017) showed that

$$n^{1/2}(\widehat{\boldsymbol{\beta}} - \boldsymbol{\beta}) = -\mathbf{A}^{-1}n^{1/2}\mathbf{U}_n(\boldsymbol{\beta}) + o_p(1), \tag{5}$$

where $\mathbf{A}$ is the asymptotic slope matrix of $\mathbf{U}_n(\boldsymbol{\beta})$. $\mathbf{A}$ can be estimated using a similar method to that for estimating $\mathbf{B}$. Specifically, we carry out the following steps:

Step I: Generate $\widetilde{L}$, say 10,000, independent realizations of $\mathbf{S} \sim N(\mathbf{0}, \mathbf{I}_q)$, denoted by $\mathbf{S}_1, \ldots, \mathbf{S}_{\widetilde{L}}$.

Step II: Calculate $n^{1/2}\mathbf{U}_n(\widehat{\boldsymbol{\beta}} + n^{-1/2}\mathbf{S}_l)$ $(l = 1, \ldots, \widetilde{L})$.

Step III: For $j = 1, \ldots, q$, regress $n^{1/2}U_{nj}(\widehat{\boldsymbol{\beta}} + n^{-1/2}\mathbf{S}_l)$ onto $\mathbf{S}_l$ $(l = 1, \ldots, \widetilde{L})$ to estimate $\mathbf{A}_j$, the j-th row of $\mathbf{A}$, using the least squares estimation.

Denote the estimator of $\mathbf{A}$ by $\widehat{\mathbf{A}}$. Combining (2) and (5), we have

$$\mathbf{E}_1(\widetilde{\mathbf{M}} - \widehat{\mathbf{M}}) = -\mathbf{B}\mathbf{A}^{-1}nU_n(\boldsymbol{\beta}) + o_p(n^{1/2}). \tag{6}$$

Let $M_i(t) = N_i(\boldsymbol{\beta}, t) - \int_{-\infty}^{t}\nu_i(\boldsymbol{\beta}, u)Y_i(\boldsymbol{\beta}, u)d\Lambda_\varepsilon(u)$, which is a martingale under the null. It is easy to show that

$$\begin{aligned}\mathbf{U}_n(\boldsymbol{\beta}) &= \frac{1}{n}\sum_{i=1}^{n}\int_{-\infty}^{\infty}\left\{\mathbf{Z}_i - \frac{\sum_{j=1}^{n}\mathbf{Z}_j\nu_j(\boldsymbol{\beta},t)Y_j(\boldsymbol{\beta},t)}{\sum_{j=1}^{n}\nu_j(\boldsymbol{\beta},t)Y_j(\boldsymbol{\beta},t)}\right\}dM_i(\boldsymbol{\beta},t) \\ &= \frac{1}{n}\mathbf{Z}^T\int_{-\infty}^{\infty}(\mathbf{I}_n - \mathbf{V}(\boldsymbol{\beta},t)\mathbf{1}^T)d\mathbf{M}(t), \end{aligned} \tag{7}$$

where $\mathbf{1}$ is a n-dimension vector of 1's, $\mathbf{Z} = (\mathbf{Z}_1, \ldots, \mathbf{Z}_n)^T$, $\mathbf{V}(\boldsymbol{\beta}, t) = (\nu_1(\boldsymbol{\beta}, t)Y_1(\boldsymbol{\beta}, t), \ldots, \nu_1(\boldsymbol{\beta}, t)Y_n(\boldsymbol{\beta}, t))^T\{\sum_{j=1}^{n}\nu_j(\boldsymbol{\beta}, t)Y_j(\boldsymbol{\beta}, t)\}^{-1}$, and $\mathbf{M}(t) = (M_1(t), \ldots, M_n(t))^T$. It is also easy to show that

$$\widehat{\mathbf{M}} = \int_{-\infty}^{\infty}(\mathbf{I}_n - \mathbf{V}(\boldsymbol{\beta},t)\mathbf{1}^T)d\mathbf{M}(t). \tag{8}$$

Combining (6), (7) and (8) , we have

$$\begin{aligned}\mathbf{E}_1\widetilde{\mathbf{M}} &= \mathbf{E}_1\widehat{\mathbf{M}} + \mathbf{E}_1(\widetilde{\mathbf{M}} - \widehat{\mathbf{M}}) \\ &= \int_{-\infty}^{\infty}(\mathbf{E}_1 - \mathbf{B}\mathbf{A}^{-1}\mathbf{Z}^T)(\mathbf{I}_n - \mathbf{V}(\boldsymbol{\beta},t)\mathbf{1}^T)d\mathbf{M}(t) + o_p(n^{1/2}) \quad (9)\end{aligned}$$

Since $\mathbf{M}(t)$ is a vector of independent martingales under the null, whose compensators are $\boldsymbol{\Lambda}(t) = \int_{-\infty}^{t} d\boldsymbol{\Lambda}(t) \equiv (\int_{-\infty}^{t} d\Lambda_1(t), \ldots, \int_{-\infty}^{t} d\Lambda_n(t))^T$, where $d\Lambda_i(t) = \nu_i(\boldsymbol{\beta},t)Y_i(\boldsymbol{\beta},t)d\Lambda_\varepsilon(t)$ $(i = 1, \ldots, n)$, we have by the martingale theory (Fleming & Harrington, 1991, Chapters 2 and 5) that

$$Cov(\mathbf{E}_1\widetilde{\mathbf{M}}) \approx \int_{-\infty}^{\infty}(\mathbf{E}_1 - \mathbf{B}\mathbf{A}^{-1}\mathbf{Z}^T)(\mathbf{I}_n - \mathbf{V}(\boldsymbol{\beta},t)\mathbf{1}^T)\mathrm{diag}(d\boldsymbol{\Lambda}(t))(\mathbf{I}_n - \mathbf{1}\mathbf{V}(\boldsymbol{\beta},t)^T)(\mathbf{E}_1^T - ZA^{-T}\mathbf{B}^T), \quad (10)$$

where diag($\mathbf{a}$) represents a diagonal matrix with $\mathbf{a}$ as the diagonal, and that $\mathbf{E}_1\widetilde{\mathbf{M}}$ is approxmiately multivariate normal with mean zero. An estimator of $Cov(\mathbf{E}_1\widetilde{\mathbf{M}})$ is

$$\widehat{Cov}(\mathbf{E}_1\widetilde{\mathbf{M}}) = \int_{-\infty}^{\infty}(\mathbf{E}_1 - \widehat{\mathbf{B}}\widehat{\mathbf{A}}^{-1}\mathbf{Z}^T)(\mathbf{I}_n - \mathbf{V}(\widehat{\boldsymbol{\beta}},t)\mathbf{1}^T)\mathrm{diag}(d\widetilde{\boldsymbol{\Lambda}}(t))(\mathbf{I}_n - \mathbf{1}\mathbf{V}(\widehat{\boldsymbol{\beta}},t)^T)(\mathbf{E}_1^T - \mathbf{Z}\widehat{\mathbf{A}}^{-T}\widehat{\mathbf{B}}^T), \quad (11)$$

where $d\widetilde{\boldsymbol{\Lambda}}(t) = (d\widetilde{\Lambda}_1(t), \ldots, d\widetilde{\Lambda}_n(t))^T$ and
$d\widetilde{\Lambda}_i(t) = \nu_i(\widehat{\boldsymbol{\beta}},t)Y_i(\widehat{\boldsymbol{\beta}},t)\sum_{j=1}^{n} dN_j(\widehat{\boldsymbol{\beta}},t)\{\sum_{j=1}^{n}\nu_j(\widehat{\boldsymbol{\beta}},t)Y_j(\widehat{\boldsymbol{\beta}},t)\}^{-1}$ $(i = 1, \ldots, n)$. By algebra, we can simplify $\widehat{Cov}(\mathbf{E}_1\widetilde{\mathbf{M}})$ as

$$\widehat{Cov}(\mathbf{E}_1\widetilde{\mathbf{M}}) = \int_{-\infty}^{\infty}(\mathbf{E}_1 - \widehat{\mathbf{B}}\widehat{\mathbf{A}}^{-1}\mathbf{Z}^T)\{\mathrm{diag}(d\widetilde{\boldsymbol{\Lambda}}(t)) - \mathbf{V}(\widehat{\boldsymbol{\beta}},t)\mathbf{1}^T d\mathbf{N}(\widehat{\boldsymbol{\beta}},t)\mathbf{V}(\widehat{\boldsymbol{\beta}},t)^T\}(\mathbf{E}_1^T - \mathbf{Z}\widehat{\mathbf{A}}^{-T}\widehat{\mathbf{B}}^T), \quad (12)$$

where $\mathbf{N}(\widehat{\boldsymbol{\beta}},t) = (N_1(\widehat{\boldsymbol{\beta}},t), \ldots, N_n(\widehat{\boldsymbol{\beta}},t))^T$. Take an eigendecomposition of $\widehat{Cov}(\mathbf{E}_1\widetilde{\mathbf{M}})$,

$$\widehat{Cov}(\mathbf{E}_1\widetilde{\mathbf{M}}) = \Gamma \begin{bmatrix}\lambda_1 & & \\ & \ddots & \\ & & \lambda_m\end{bmatrix} \Gamma^T, \quad (13)$$

where Γ is a $m \times m$ orthogonal matrix. Together with the asymptotic normality of $\mathbf{E}_1\widetilde{\mathbf{M}}$,

we have

$$\mathbf{E}_1\widetilde{\mathbf{M}} \overset{\mathrm{d}}{\approx} \Gamma \begin{bmatrix} \lambda_1^{1/2} & & \\ & \ddots & \\ & & \lambda_m^{1/2} \end{bmatrix} \boldsymbol{\chi}, \tag{14}$$

where $\boldsymbol{\chi} \equiv (\chi_{11}, \ldots, \chi_{1m})^T \sim N(\mathbf{0}, \mathbf{I}_m)$. Thus,

$$R = \widetilde{\mathbf{M}}^T \mathbf{K} \widetilde{\mathbf{M}} = \widetilde{\mathbf{M}}^T \mathbf{E}_1^T \mathbf{E}_1 \widetilde{\mathbf{M}} \overset{\mathrm{d}}{\approx} \sum_{j=1}^{m} \lambda_j \chi_{1j}^2, \tag{15}$$

where χ_{1j}^2's are independent chi-square variables with degree 1.

B Additional Simulations

In the simulations of Sections B.1, B.2, B.3 and B.6, we sampled SNPs from the genotype data of the 1000 Genomes Project (phase 3) (1000 Genomes Project Consortium et al., 2015) as the genetic markers. In the other simulations, a two-step procedure was used to simulate SNPs as the genetic markers under testing: 1) sample n vectors independently from a multivariate normal distribution with a zero mean and the covariance matrix being $\mathbf{\Sigma}_{p\times p} = \{0.5^{|k-l|}\}$; 2) categorize each component of every multivariate normal vector into three levels labeled with 0, 1 and 2 using the cut-off values that were selected to satisfy the Hardy-Weinberg equilibrium (HWE) and the minor allele frequency (MAF) simulated from $Beta(2, 5)$. Left-truncation times and censoring times were generated as in the main text.

B.1 Testing G-G/G-E interaction

In this set of simulations, we compared the performances of the test R_{int} and the regular Wald test in testing the interaction between **G**, a SNP set, and **H**, a SNP set or a two dimensional dummy variable created based on a three-categorical variable acting as an environmental factor. Unlike the proposed association tests, the interaction tests involve fitting a null model that parametrically models the genetic main effect(s). As a result, with a large number of genetic markers, the asymptotic normality of the rank-based parameter estimator (Chiou & Xu, 2017) for the AFT null model may not hold under the null unless

the sample size is very large, making the Type I errors of the interaction tests out of control. Therefore, we reduced the marker set sizes and increased the sample sizes in the simulations of G-G/G-E interactions. For the G-G interaction scenario, we considered $p = q = 5,\ 6$ and $n = 2000,\ 2300$. For the G-E interaction scenario, we considered $p = 6,\ 9,\ q = 2$ and $n = 1600,\ 1800$. Larger sample sizes were used in the simulation of G-G interaction because the considered null models have more covariates. We centered the SNP covariates before performing the proposed interaction tests and the Wald test, as explained in Section 2.2. The Wald test was based on the rank-based estimator (Chiou & Xu, 2017) for the coefficients of the interaction terms and its asymptotic normal distribution, derived by Chiou and Xu (2017).

The cause-specific hazard functions in the simulation of G-G interaction were:

$$\lambda_1(t|\mathbf{G},\mathbf{H}) = \lambda_0(t\cdot\exp\{-\mathbf{G}^T\mathbf{0.05}-\mathbf{H}^T\mathbf{0.05}-(\mathbf{GH})^T\boldsymbol{\beta}\})\exp\{-\mathbf{G}^T\mathbf{0.05}-\mathbf{H}^T\mathbf{0.05}-(\mathbf{GH})^T\boldsymbol{\beta}\}, \tag{16}$$

$$\lambda_2(t|\mathbf{G},\mathbf{H}) = \lambda_0(t\cdot\exp\{-\mathbf{G}^T\mathbf{0.2}-\mathbf{H}^T\mathbf{0.2}\})\exp\{-\mathbf{G}^T\mathbf{0.2}-\mathbf{H}^T\mathbf{0.2}\}, \tag{17}$$

where **GH** denotes the vector of the pair-wise products between the elements of **G** and those of **H**, **0.05** and **0.2** are p- or q-dimensional vectors with all elements equal to 0.05 or 0.2, and $\boldsymbol{\beta} = (\beta_1, \ldots, \beta_{pq-1}, \beta_{pq})^T$. We set $\boldsymbol{\beta} = (0, \ldots, 0)^T$ under the null hypothesis of no **G-H** interaction, while $\boldsymbol{\beta} = (0.1, \ldots, 0.1)^T$ under the alternative hypothesis that the interaction exists. The linear kernel was used to measure the genetic similarity in the G-G interaction test.

The cause-specific hazard functions in the simulation of G-E interaction were:

$$\lambda_1(t|\mathbf{G},\mathbf{H}) = \lambda_0(t\cdot\exp\{-\mathbf{G}^T\mathbf{0.2}-\mathbf{H}^T\mathbf{0.2}-(\mathbf{GH})^T\boldsymbol{\beta}\})\exp\{-\mathbf{G}^T\mathbf{0.2}-\mathbf{H}^T\mathbf{0.2}-(\mathbf{GH})^T\boldsymbol{\beta}\}, \tag{18}$$

$$\lambda_2(t|\mathbf{G},\mathbf{H}) = \lambda_0(t\cdot\exp\{-\mathbf{G}^T\mathbf{0.5}-\mathbf{H}^T\mathbf{0.5}\})\exp\{-\mathbf{G}^T\mathbf{0.5}-\mathbf{H}^T\mathbf{0.5}\}. \tag{19}$$

We set $\boldsymbol{\beta} = (0, \ldots, 0)^T$ under the null hypothesis of no **G-H** interaction, while

$\boldsymbol{\beta} = (0.15, \ldots, 0.15)^T$ under the alternative hypothesis that the interaction exists. The linear kernel was used to measure the genetic similarity and the identity kernel was used to measure the environmental similarity in the G-E interaction test.

Web Table 1 shows that the empirical sizes of R_{int} are close to the nominal level, whereas those of the Wald tests are inflated. When testing the G-G interaction, R_{int} is more powerful than the Wald test. This power advantage in the presence of LD between the SNPs of interest was also observed in some other kernel-based multi-marker interaction tests (Li, Wu, & Lu, 2021; Wu, Li, & Lu, 2021). Although R_{int} exhibits lower power than the Wald test in detecting G-E interactions in the considered settings, this is likely because of the Wald test's anti-conservative behavior under the given p, q and n, as shown by the inflated empirical sizes. We have also conducted G-E interaction simulations under $p = 2, 3$, $q = 1$ and $n = 500, 900$. In these scenarios, the empirical sizes of R_{int} and the Wald test are both close to the nominal level, and R_{int} is more powerful than the Wald test. The detailed results are omitted here due to space limitations.

B.2 Small-sample adjustment

In this series of simulations, we assess the performances of the small-sample corrected tests proposed in Section 2.3 by comparing the empirical sizes and powers of: 1) R^C and R in testing genetic association in the absence of genetic heterogeneity, 2) R^c_{het} and R_{het} in testing genetic association in the presence of genetic heterogeneity across two observable sub-populations, and 3) R^c_{int} and R_{int} in testing G-G/G-E interaction. We use small n relative to p (and q).

B.2.1 Empirical sizes and powers of R and R^c. In this simulation, we investigated the performances of R and R^c in testing genetic association in the absence of genetic heterogeneity. The simulation setting is similar to that for checking R's performance in testing association under no genetic heterogeneity and no confounding in the main text. The setting changes are the following. We decreased the sample size to $n = 100$ and set

$p = 15$. The cause-specific hazard functions were

$$\lambda_1(t|\mathbf{Z}, \mathbf{G}) = \lambda_0(t \cdot \exp\{-\sum_{j=1}^{p} \beta_j G_j - \sum_{k=1}^{2} 0.1 Z_k\}) \exp\{-\sum_{j=1}^{p} \beta_j G_j - \sum_{k=1}^{2} 0.1 Z_k\} \tag{20}$$

and

$$\lambda_2(t|\mathbf{Z}, \mathbf{G}) = \lambda_0(t \cdot \exp\{-\sum_{j=1}^{p} 0.2 G_j - \sum_{k=1}^{2} 0.2 Z_k\}) \exp\{-\sum_{j=1}^{p} 0.2 G_j - \sum_{k=1}^{2} 0.2 Z_k\}, \tag{21}$$

where $\beta_j = 0.1$ and $\beta_j = 0$ ($j = 1, \ldots, p$) in the power evaluation and size assessment respectively. The log-scale uniform Q-Q plots in Web Figure 1 show that the null distribution of R^c's p-value is closer to $U(0, 1)$ than that of R's p-value under small samples. The comparison of the size and power of R and R^c is shown in Web Table 8. The results indicate that when the sample size is small, the test R becomes conservative, but R^c still controls Type I error well and is more powerful than R.

To evaluate the performance of the tests R and R^c when applied to high-dimensional markers, we performed a simulation with $n = 1500$ and $p = 1500$. In this simulation, the cause-specific hazard functions were

$$\lambda_1(t|\mathbf{Z}, \mathbf{G}) = \lambda_0(t \cdot \exp\{-\sum_{j=1}^{p} \beta_j G_j - \sum_{k=1}^{2} 0.1 Z_k\}) \exp\{-\sum_{j=1}^{p} \beta_j G_j - \sum_{k=1}^{2} 0.1 Z_k\} \tag{22}$$

and

$$\lambda_2(t|\mathbf{Z}, \mathbf{G}) = \lambda_0(t \cdot \exp\{-\sum_{j=1}^{p} 0.01 G_j - \sum_{k=1}^{2} 0.2 Z_k\}) \exp\{-\sum_{j=1}^{p} 0.01 G_j - \sum_{k=1}^{2} 0.2 Z_k\}, \tag{23}$$

where $\beta_j = 0.005$ and $\beta_j = 0$ ($j = 1, \ldots, p$) in the power evaluation and size assessment respectively. Web Table 9 and Web Figure 2 show that both R and R^c are able to control Type I error rates well while R^c has slightly higher power.

B.2.2 Empirical sizes and powers of $R_{\textbf{het}}$ and $R^c_{\textbf{het}}$. In this simulation, we investigated the performances of R_{het} and R^c_{het} in testing genetic association under genetic heterogeneity across two observable sub-populations. We set $n = 200$ and $p = 20$. The cause-specific hazard functions were:

$$\lambda_1(t|Z_i, \mathbf{G}_i) = \lambda_0(t \cdot \exp\{-\sum_{j=1}^{p} (\beta_0 + \beta_1 Z_i) G_{ij} - 0.5 Z_i\}) \exp\{-\sum_{j=1}^{p} (\beta_0 + \beta_1 Z_i) G_{ij} - 0.5 Z_i\} \tag{24}$$

and

$$\lambda_2(t|Z_i, \mathbf{G}_i) = \lambda_0(t \cdot \exp\{-\sum_{j=1}^{p} 0.35 G_{ij} - Z_i\}) \exp\{-\sum_{j=1}^{p} 0.35 G_{ij} - Z_i\}, \tag{25}$$

where Z_i is the sex variable in the 1000 Genomes (phase 3) data set. To assess the size and the power of R_{het} and R^c_{het}, we set $\beta_0 = 0$ and $\beta_0 = 0.002$, respectively. We also set $\beta_1 = 0$ in the size assessment and $\beta_1 = 0.15$ and 0.25 in the power assessment, with the larger β_1 representing the stronger genetic heterogeneity. The log-scale uniform Q-Q plots in Web Figure 3 show that the null distribution of R^c_{het}'s p-value is closer to $U(0, 1)$ than that of R_{het}'s p-value under small samples. Web Table 10 shows that when the sample size is small, the test R_{het} becomes conservative, but R^c_{het} controls Type I error well and is more powerful than R_{het}.

B.2.3 Empirical sizes and powers of R_{int} and R^c_{int}. In this simulation, we investigated the performances of R^c_{int} and R_{int} in testing G-G and G-E interactions. The simulation settings are the same as those in Section B.1 except with different values of n, p and q, specifically $n = 1000$, $p = 10$ and $q = 10$ for the G-G interaction and $n = 1000$, $p = 10$ and $q = 2$ for the G-E interaction. Web Figures 4 and 5 show that the null distribution of R^c_{int}'s p-value is closer to $U(0, 1)$ than that of R_{int}'s p-value. Web Tables 11 and 12 show that when the sample size is not sufficiently large in light of p and q, the test R_{int} becomes anti-conservative, but R^c_{int} still controls Type I error well and is just a little less powerful than R_{int}.

B.3 Empirical sizes of R, R_{het} R^c, and R^c_{het} under stringent p-value thresholds

Genome-wide association studies with genotyping or sequencing data usually test the associations between hundreds of thousands of genetic variants and a phenotype, causing a severe multiple testing problem. Common approaches to address the multiple testing issue, such as the Bonferroni correction and the Benjamini-Hochberg procedure (Benjamini & Hochberg, 1995), lead to stringent p-value thresholds when applied to those association analyses. In this simulation, we checked the sizes of the tests R, R_{het}, R^c, and R^c_{het} under

stringent p-value thresholds (i.e., those that are much smaller than 0.05). The simulation setting for R and R^c was the same as that for assessing R's size and power in the absence of genetic heterogeneity under the 0.05 level. The simulation setting for R_{het} and R^c_{het} was the same as that for assessing R_{het}'s size and power in the presence of genetic heterogeneity across two observable sub-populations under the 0.05 level. In both of the scenarios, 500K Monte Carlo samples with $n = 500$ and $p = 20$ were generated to calculate the empirical sizes. Web Table 2 shows that the empirical sizes of R^c and R^c_{het} are very close to the stringent p-value thresholds. Those of R and R_{het} are close to the nominal level under the threshold of 0.05, but the two tests became conservative under the smaller thresholds, which was probably due to the relatively small sample size to the number of markers. When we increased n to 1000, the empirical sizes of R and R_{het} aligned more closely with the nominal level under the stringent p-value thresholds (Web Table 13). These results indicate that the proposed association tests, especially R^c and R^c_{het}, are suitable for large-scale genetic association analyses.

B.4 Empirical size and power of R_{het} in the presence of genetic heterogeneity across two latent sub-populations

In this simulation, we investigated the empirical size and power of R_{het} as well as R under genetic heterogeneity across two latent sub-populations with equal proportions. The survival time of the j-th subject in the i-th sub-population was generated from the following AFT models for the cause-specific hazard functions,

$$\lambda_1(t|\mathbf{Z}_{ij}, \mathbf{G}_{ij}) = \lambda_0(t \cdot \exp\{-\sum_{k=1}^{p} G_{ijk}\beta_{ik} - 0.1Z_{ij1} - 0.1Z_{ij2}\}) \exp\{-\sum_{k=1}^{p} G_{ijk}\beta_{ik} - 0.1Z_{ij1} - 0.1Z_{ij2}\}, \tag{26}$$

$$\lambda_2(t|\mathbf{Z}_{ij}, \mathbf{G}_{ij}) = \lambda_0(t \cdot \exp\{-\sum_{k=1}^{p} 0.02G_{ijk} - 0.2Z_{ij1} - 0.2Z_{ij2}\}) \exp\{-\sum_{k=1}^{p} 0.02G_{ijk} - 0.2Z_{ij1} - 0.2Z_{ij2}\}, \tag{27}$$

where $\beta_{i1} = \ldots = \beta_{ip}$ $(i = 1, 2)$ represent the effects of G_k's $(k = 1, \ldots, p)$ in Sub-population i and vary depending on the heterogeneity scenario. A continuous variable, X_{ij} $(i = 1, 2)$, was simulated to infer the sub-population. Specifically, $X_{ij} = I(i = 1) + 1 + e_{ij}$, where $e_{ij} \sim N(0, 0.5)$. We set $\beta_{ik} = 0$ $(i = 1, 2;\ k = 1, \ldots, p)$ in the size assessment, and different values were assigned to β_{ik}'s to represent different heterogeneity scenarios in the power evaluation, as shown in Web Table 5. The IBS kernel was used to measure the genetic similarity in R_{het} and R, and the Gaussian kernel was applied to X_{ij} to measure the sub-population similarity in R_{het}. Web Table 4 shows that the empirical sizes of both R_{het} and R are close to the nominal level. Web Table 5 shows that the power of R_{het} increases with the sample size and the heterogeneity size, measured by $|\beta_{1k} - \beta_{2k}|$, the genetic effect difference between the two sub-populations. Also, Web Table 5 indicates that when there is no genetic heterogeneity (Scenario T1) , R_{het} has a smaller power than R. However, R_{het} is more powerful when there exists genetic heterogeneity between the two latent sub-populations (Scenarios T2 - T4). We set p to be smaller in the power simulations than in the size simulations to prevent Web Table 5 from containing many powers of one. The power pattern will stay the same when p is large.

B.5 Empirical size and power of R_{het} in the presence of genetic heterogeneity across individual genome profiles

In this simulation, we investigated the performances of R_{het} and R when the sub-population structure is "continuous". Specifically, we let the genetic effect vary across individual genome profiles instead of a small number of sub-populations, e.g., males and females. The survival time of the i-th $(i = 1, \ldots, n)$ subject was generated from the models (26) and (27), where $\beta_{i1} = \beta_{i2} = \ldots = \beta_{ip} = 0$ in the size assessment and were randomly sampled from a uniform distribution with mean μ_β and variance σ^2_β in the power assessment. The values of μ_β and σ^2_β vary in different simulation scenarios. We simulated a set of 1000 SNPs for each subject, $\{X_{id}\}_{d=1}^{1000}$ $(i = 1, \ldots, n)$, to represent the genome profile.

For each $1 \leq d \leq 1000$, $\mathbf{X}_d = (X_{1d}, \ldots, X_{nd})^T$ was generated in two steps: 1) sample $\tilde{\mathbf{X}}_d = (\widetilde{X}_{1d}, \ldots, \widetilde{X}_{nd})^T$ from a multivariate normal distribution, $MVN(\mathbf{0}, \mathbf{\Sigma})$, where $\mathbf{\Sigma}$ is an $n \times n$ covariance matrix with the (i,j)-th element being $\Sigma_{ij} = I(i = j)$ under the null hypothesis (i.e., no genetic association) and $\Sigma_{ij} = \exp(-|\beta_{i1} - \beta_{j1}|/\sigma_\beta)$ under the alternative; 2) $\mathbf{X}_d$ is then obtained by categorizing each element of $\widetilde{\mathbf{X}}_d$ into three levels, 0, 1 and 2, using rank-based cut-off values selected to achieve the Hardy-Weinberg equilibrium and a pre-specified minor allele frequency that was randomly sampled from $Beta(1, 3)$. We used the IBS kernel to measure both the genetic and the sub-population similarities in R_{het} and R. Web Table 6 shows that the empirical sizes of both tests are around the nominal level. Web Table 7 shows that R_{het} is more powerful than R under genetic heterogeneity across individual genome profiles, and the power advantage increases with the genetic heterogeneity size (σ_β).

B.6 Comparing sizes and powers of R and coxKM under an AFT data-generating model with four adjustment covariates

In this simulation, we evaluated the empirical sizes and powers of our test R and the Cox model-based test coxKM (Cai, Tonini, & Lin, 2011) in testing genetic association when data are generated from an AFT model that has two more adjustment covariates than the one for Table 3. Specifically, the survival time of Subject i ($i = 1, \ldots, n$) was generated from the following AFT models for the cause-specific hazard functions,

$$\lambda_1(t|\mathbf{Z}_i, \mathbf{G}_i) = \lambda_0(t \cdot \exp\{-\sum_{j=1}^{p} \beta_j G_{ij} - 0.1Z_{i1} + 0.1Z_{i2} - 0.1Z_{i3} + 0.1Z_{i4} - 0.7\}) \cdot \exp\{-\sum_{j=1}^{p} \beta_j G_{ij} - 0.1Z_{i1} + 0.1Z_{i2} - 0.1Z_{i3} + 0.1Z_{i4} - 0.7\} \tag{28}$$

and

$$\lambda_2(t|\mathbf{Z}_i, \mathbf{G}_i) = \lambda_0(t \cdot \exp\{-\sum_{j=1}^{p} 0.16G_{ij} - 1.5Z_{i1} + 1.5Z_{i2} - 1.5Z_{i3} + 1.5Z_{i4}\})\cdot$$
$$\exp\{-\sum_{j=1}^{p} \beta_j G_{ij} - 1.5Z_{i1} + 1.5Z_{i2} - 1.5Z_{i3} + 1.5Z_{i4}\}, \quad (29)$$

where $\beta_j = 0.08$ and $\beta_j = 0$ $(j = 1, \ldots, p)$ in the power evaluation and size assessment respectively. Z_{i1} is a Bernoulli random variable with success probability 0.6, $Z_{i2} \sim Unif(0, 2)$, Z_{i3} and Z_{i4} are two continuous random variables that follow $N(0, 1)$ distribution. Web Table 14 shows that the empirical size of R is close to the nominal level and R is much more powerful than coxKM. In contrast, coxKM is very conservative when applied to survival data from AFT models. Web Figure 6 shows that R's p-value follows the $U[0, 1]$ distribution under the null, whereas the null distribution of coxKM's p-value deviates from the uniform distribution.

B.7 Runtimes of the proposed interaction tests and the small-sample adjusted versions of all the new tests

We measured the runtimes of the proposed interaction tests and all the small-sample adjusted tests. For comparison with the unadjusted tests, the runtimes of R and R_{het} were measured again. The runtime measurements of the association tests R, R_{het}, R^c and R^c_{het} were taken in the settings of Web Table 14, and those of the interaction tests R_{int} and R^c_{int} were taken in the setting of Web Table 11 except that more combinations of n, p and q were considered. The computing environment was the same as for the runtime simulations in the main text. Web Tables 15 and 16 show the average runtimes of the association tests and the interaction tests, respectively. An interesting observation is that the number of genetic markers affects the runtimes of the interaction tests, but it does not affect those of the association tests as much. This observation suggests that fitting the null model is the most time-consuming part of all the proposed tests.

References

1000 Genomes Project Consortium, et al. (2015). A global reference for human genetic variation. *Nature*, *526*(7571), 68-74.

Benjamini, Y., & Hochberg, Y. (1995). Controlling the false discovery rate: A practical and powerful approach to multiple testing. *Journal of the Royal Statistical Society. Series B (Methodological)*, *57*(1), 289–300.

Cai, T., Tonini, G., & Lin, X. (2011). Kernel machine approach to testing the significance of multiple genetic markers for risk prediction. *Biometrics*, *67*(3), 975-986.

Chiou, S. H., & Xu, G. (2017). Rank-based estimation for semiparametric accelerated failure time model under length-biased sampling. *Statistics and Computing*, *27*(2), 483–500.

Fleming, T. R., & Harrington, D. P. (1991). *Counting processes and survival analysis.* John Wiley & Sons.

Lai, T. L., & Ying, Z. (1991). Rank Regression Methods for Left-Truncated and Right-Censored Data. *The Annals of Statistics*, *19*(2), 531 – 556.

Li, C., Wu, D., & Lu, Q. (2021). Set-based genetic association and interaction tests for survival outcomes based on weighted v statistics. *Genetic Epidemiology*, *45*, 46-63.

Wu, D., Li, C., & Lu, Q. (2021). Multi-marker genetic association and interaction tests with interval-censored survival outcomes. *Genetic Epidemiology*, *45*(8), 860-873.

Zeng, D., & Lin, D. Y. (2008). Efficient resampling methods for nonsmooth estimating functions. *Biostatistics*, *9*(2), 355-363.

Web Table 1

Empirical sizes and powers of R_{int} and the Wald test in testing G-G/G-E interaction under left truncation.

	Empirical Size (Power) in testing G-G interaction	
	p=q=5, n=2000	p=q=5, n=2300
R_{int}	0.059 (0.771)	0.052 (0.847)
Wald	0.081 (0.536)	0.073 (0.605)
	p=q=6, n=2000	p=q=6, n=2300
R_{int}	0.051 (0.870)	0.057 (0.906)
Wald	0.090 (0.614)	0.081 (0.677)
	Empirical Size (Power) in testing G-E interaction	
	p=6, q=2, n=1600	p=6, q=2, n=1800
R_{int}	0.058 (0.550)	0.051 (0.608)
Wald	0.072 (0.628)	0.061 (0.672)
	p=9, q=2, n=1600	p=9, q=2, n=1800
R_{int}	0.054 (0.663)	0.051 (0.714)
Wald	0.078 (0.690)	0.074 (0.761)

Web Table 2

Empirical sizes of R, R_{het}, R^c and R^c_{het} under stringent p-value thresholds and left truncation, with $n = 500$ and $p = 20$.

	Empirical Size			
Threshold	R	R_{het}	R^c	R^c_{het}
0.05	0.045	0.041	0.051	0.051
0.005	0.0037	0.0033	0.0051	0.0053
0.0005	0.00028	0.00023	0.00052	0.00058
0.00005	0.000026	0.000012	0.000050	0.000050

Web Table 3

Empirical size and power of R in testing genetic effects under quadratic confounding and left truncation.

Empirical Size (Power)	
p=20, n=400	p=20, n=500
0.056 (0.771)	0.052 (0.863)
p=25, n=400	p=25, n=500
0.046 (0.816)	0.052 (0.890)

Web Table 4

Empirical sizes of R and R_{het} in testing genetic effects under genetic heterogeneity across two latent sub-populations, covariate adjustment and left truncation.

	Empirical Size	
	p=20, n=400	p=20, n=500
R_{het}	0.047	0.050
R	0.055	0.052
	p=25, n=400	p=25, n=500
R_{het}	0.053	0.050
R	0.044	0.048

Web Table 5

Powers of R and R_{het} in testing genetic effects under genetic heterogeneity across two latent sub-populations, covariate adjustment and left truncation. Various heterogeneity scenarios were considered, determined by the values of β_{1k} and β_{2k}, including the same effect size and the same effect direction (T1), identical sizes but opposite directions (T2), no effect in one sub-population while positive effect in the other (T3), and different sizes but the same direction (T4).

		Heterogeneity Scenario							
		T1		T2		T3		T4	
	β_{1k}	0.04	0.08	-0.05	-0.1	0	0	0.03	0.03
	β_{2k}	0.04	0.08	0.05	0.1	0.08	0.12	0.08	0.1
p=3,n=400	R_{het}	0.252	0.768	0.775	0.972	0.719	0.906	0.601	0.784
	R	0.376	0.891	0.056	0.043	0.370	0.636	0.596	0.746
p=3,n=500	R_{het}	0.327	0.825	0.826	0.979	0.768	0.945	0.703	0.868
	R	0.376	0.927	0.057	0.047	0.467	0.716	0.702	0.817
p=5,n=400	R_{het}	0.403	0.900	0.984	1.000	0.950	0.994	0.855	0.958
	R	0.594	0.976	0.062	0.069	0.557	0.775	0.829	0.894
p=5,n=500	R_{het}	0.467	0.968	0.988	1.000	0.968	0.998	0.935	0.979
	R	0.729	0.995	0.047	0.068	0.659	0.866	0.905	0.958

Web Table 6

Empirical sizes of R_{het} and R in testing genetic effects under genetic heterogeneity across individual genome profiles, covariate adjustment and left truncation.

	Empirical Size	
	p=20, n=400	p=20, n=500
R_{het}	0.052	0.047
R	0.058	0.051
	p=25, n=400	p=25, n=500
R_{het}	0.045	0.048
R	0.046	0.052

Web Table 7

Powers of R_{het} and R under genetic heterogeneity across individual genome profiles, covariate adjustment and left truncation.

	Power			
	p=20, n=400		p=20, n=500	
$(\mu_\beta, \sigma_\beta)$	(0.005, 0.005)	(0.005, 0.01)	(0.005, 0.005)	(0.005, 0.01)
R_{het}	0.300	0.886	0.368	0.910
R	0.051	0.047	0.045	0.056
	p=25, n=400		p=25, n=500	
$(\mu_\beta, \sigma_\beta)$	(0.005, 0.005)	(0.005, 0.01)	(0.005, 0.005)	(0.005, 0.01)
R_{het}	0.501	0.946	0.615	0.956
R	0.048	0.057	0.047	0.057

Web Table 8

Empirical sizes and powers of R and R^c under left truncation with $n = 100$ and $p = 20$.

	Empirical Size (power)
R	0.029 (0.378)
R^c	0.052 (0.476)

Web Table 9

Empirical sizes and powers of R and R^c under left truncation with $n = 1500$ and $p = 1500$.

	Empirical Size (power)
R	0.044 (0.400)
R^c	0.051 (0.443)

Web Table 10

Empirical sizes and powers of R_{het} and R^c_{het} under genetic heterogeneity across two observable sub-populations and left truncation, with $n = 200$ and $p = 20$.

	Empirical Size	Power	
β_1	0	0.15	0.25
R_{het}	0.032	0.290	0.519
R^c_{het}	0.055	0.393	0.620

Web Table 11

Empirical sizes and powers of R_{int} and R^c_{int} in testing G-G interaction under left truncation, with $n = 1000$, $p = q = 10$.

	Empirical Size (Power)
R_{int}	0.086 (0.410)
R^c_{int}	0.052 (0.405)

Web Table 12

Empirical sizes and powers of R_{int} and R^c_{int} in testing G-E interaction under left truncation, with $n = 1000$, $p = 10$ and $q = 2$.

	Empirical Size (Power)
R_{int}	0.065 (0.892)
R^c_{int}	0.054 (0.861)

Web Table 13

Empirical sizes of R and R_{het} under stringent p-value thresholds and left truncation, with $n = 1000$ and $p = 20$.

	Empirical Size	
Threshold	R	R_{het}
0.05	0.047	0.045
0.005	0.0041	0.0040
0.0005	0.00038	0.00038
0.00005	0.00038	0.00046

Web Table 14

Empirical sizes and powers of R and coxKM with data from an AFT model having four adjustment covariates.

	Empirical Size (Power)	
	p=20, n=400	p=20, n=500
R	0.047 (0.298)	0.045 (0.367)
coxKM	0.005 (0.034)	0.001 (0.050)
	p=25, n=400	p=25, n=500
R	0.044 (0.353)	0.053 (0.453)
coxKM	0.002 (0.067)	0.001 (0.091)

Web Table 15

Runtimes of the proposed association tests in testing genetic effects in the absence of genetic heterogeneity and confounding.

	Average Runtime Based on Ten Replicates (seconds)			
	R	R_{het}	R^c	R^c_{het}
p=20, n=400	4.56	5.08	5.53	5.57
p=25, n=400	5.28	5.71	6.30	6.44
p=20, n=500	6.51	7.01	8.15	8.18
p=25, n=500	6.74	7.35	8.33	8.40

Web Table 16

Runtimes of the proposed interaction tests in testing G-G interaction effects.

	Average Runtime Based on Five Replicates (seconds)	
	R_{int}	R^c_{int}
p=q=7, n=400	17.52	15.20
p=q=10, n=400	26.38	26.34
p=q=7, n=500	23.54	23.80
p=q=10, n=500	45.53	38.07

Web Table 17

Percentiles of the SNP set size in ROSMAP genotype data.

	0%	10%	20%	30%	40%	50%	60%	70%	80%	90%	100%
Percentile	1	1	2	3	5	6	9	12	18	35	1434

Web Table 18

The genomic inflation factors (ratio of the expected to observed median p-value) for R^c *and* R^c_{het} *with the ROSMAP data. IBS, Lin, Lap and Quad stand for the IBS, linear, Laplacian and quadratic kernels, respectively. Various types of heterogeneity were considered, including no genetic heterogeneity (S1), heterogeneity between sexes (S2), heterogeneity across education attainment categories (S3), and heterogeneity across genetic backgrounds (S4).*

Genetic similarity kernel	Scenario	Genomic inflation factor
IBS	S1	1.0085
	S2	0.8624
	S3	0.9918
	S4	1.0047
Lin	S1	1.0041
	S2	0.9637
	S3	0.9939
	S4	1.0037
Lap	S1	1.0128
	S2	0.8897
	S3	0.9990
	S4	1.0096
Quad	S1	1.0040
	S2	0.9751
	S3	1.0019
	S4	1.0028

Web Table 19

Results of the interaction tests in the ROSMAP data analysis.

	APOE4-*APOC1*	*APOE4*-sex	*APOE4*-education	*APOC1*-sex	*APOC1*-education
p-value	0.426	0.971	0.374	0.257	0.166
	IGSF23-*APOE4*	*IGSF23*-*APOC1*	*IGSF23*-education	*IGSF23*-sex	
p-value	0.492	0.139	0.808	0.594	

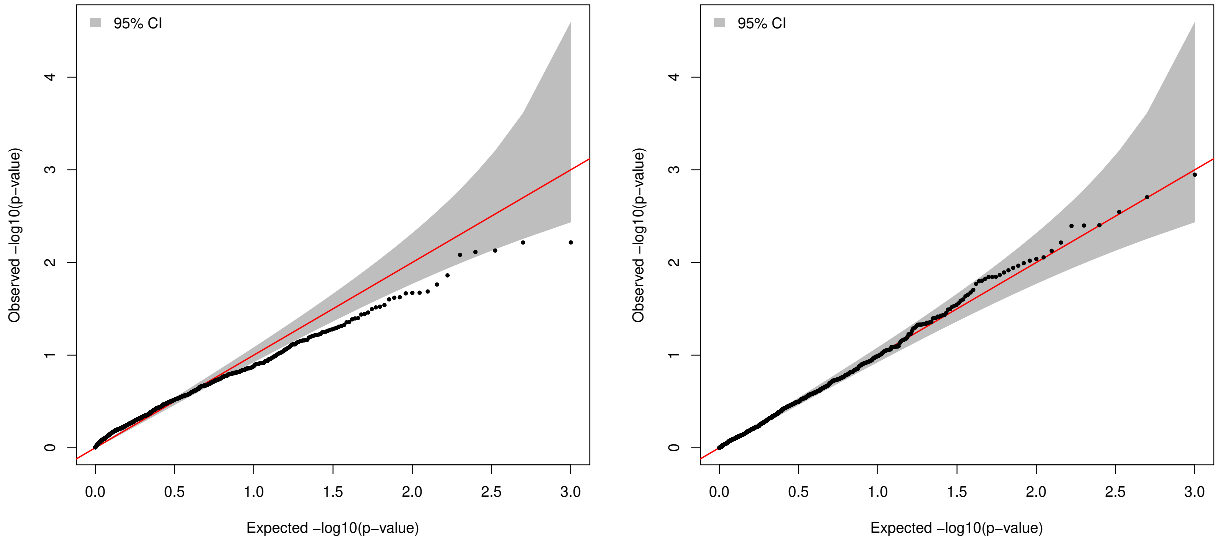

(a) R (b) R^c

Web Figure 1. Log-scale uniform Q-Q plots of the null p-value of R and R^c in testing genetic association under no genetic heterogeneity with $n = 100$ and $p = 20$.

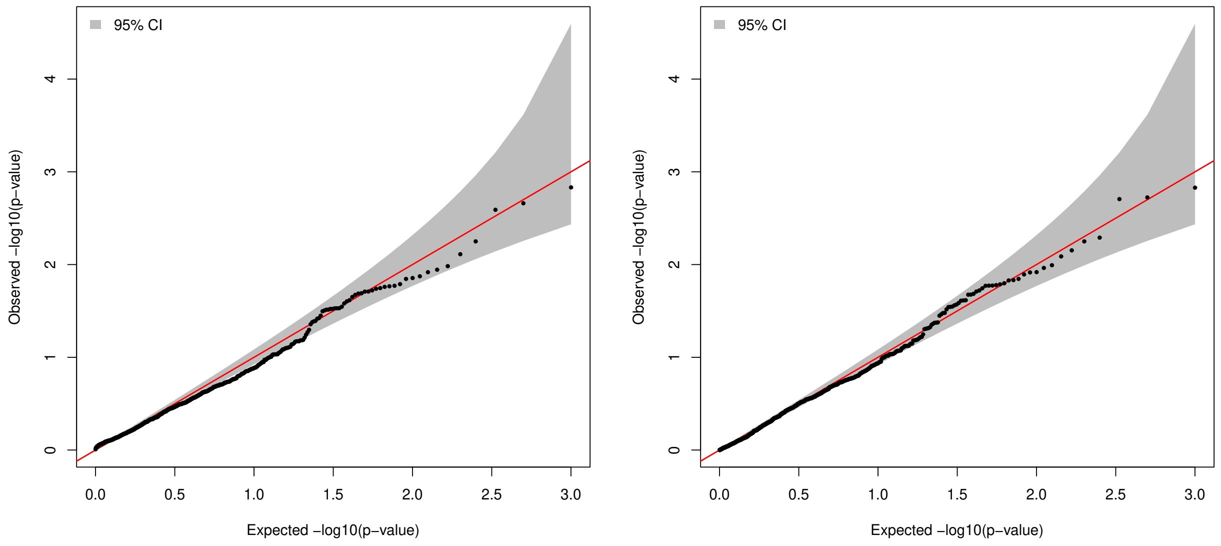

(a) R (b) R^c

Web Figure 2. Log-scale uniform Q-Q plots of the null p-value of R and R^c in testing genetic association under no genetic heterogeneity with $n = 1500$ and $p = 1500$.

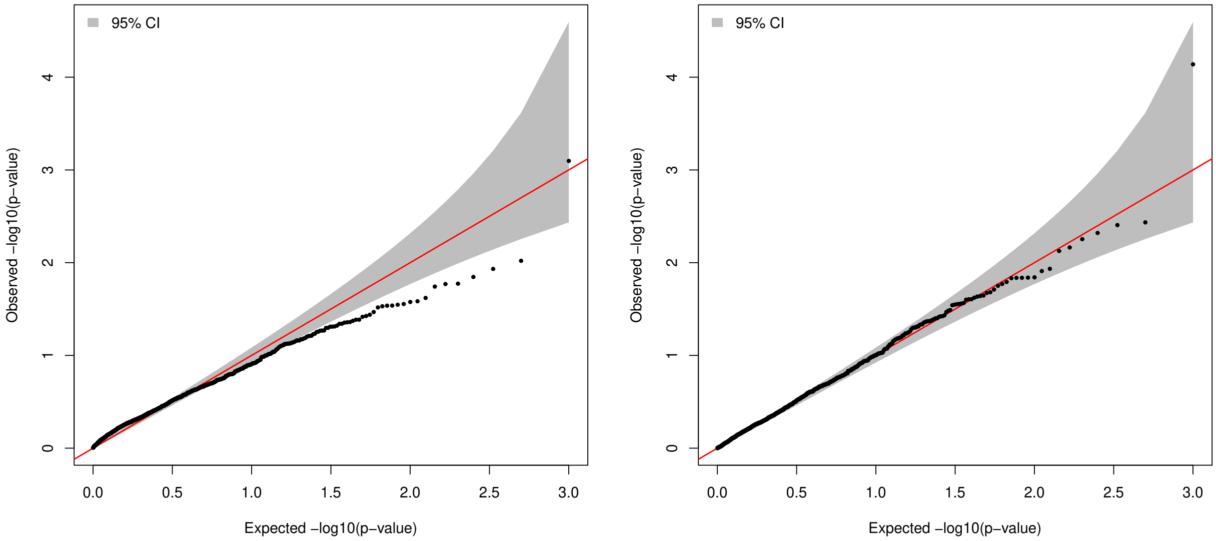

(a) R_{het} (b) R^c_{het}

Web Figure 3. Log-scale uniform Q-Q plots of the null p-values of R_{het} and R^c_{het} considering genetic heterogeneity across two observable sub-populations, with $n = 200$ and $p = 20$.

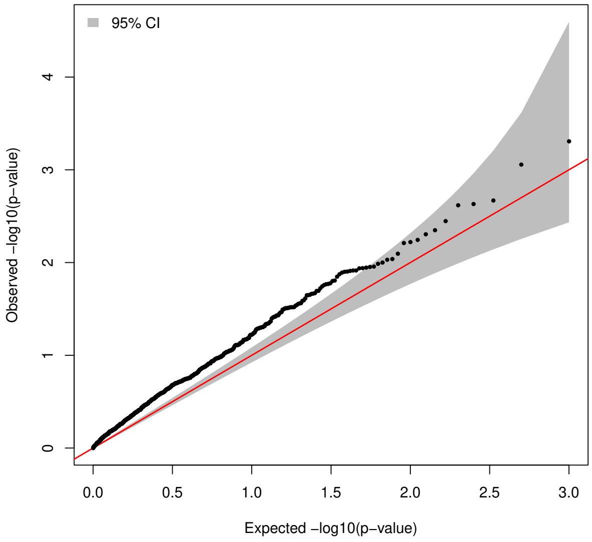

(a) R_{int}

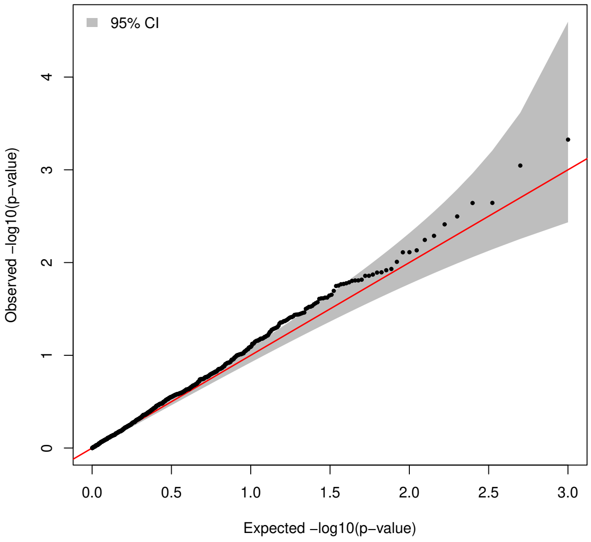

(b) R^c_{int}

Web Figure 4. Log-scale uniform Q-Q plots of the null p-values of R_{int} and R^c_{int} in testing G-G interaction, with $n = 1000,\ p = q = 10$.

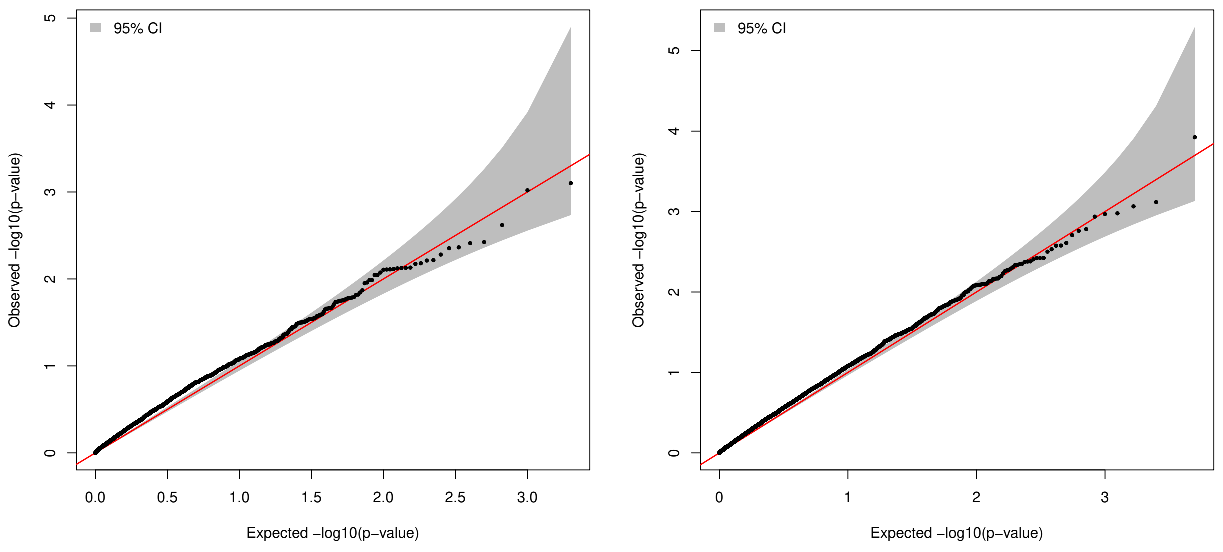

(a) R_{int} (b) R^c_{int}

Web Figure 5. Log-scale uniform Q-Q plots of the null p-values of R_{int} and R^c_{int} in testing G-E interaction, with n=1000, p=10 and q=2.

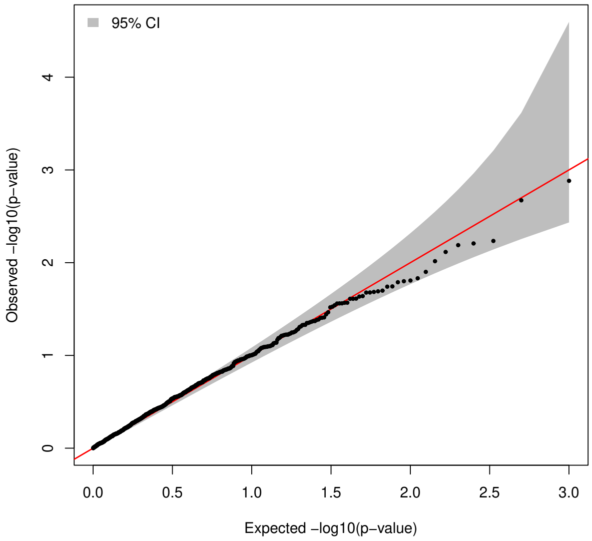

(a) R

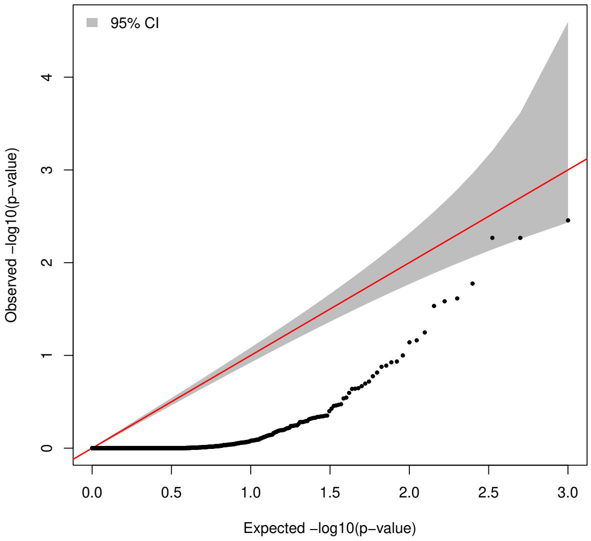

(b) coxKM

Web Figure 6. Log-scale uniform Q-Q plots of the null p-values of R and coxKM in testing genetic association when the survival data are generated from an AFT model with four adjustment covariates.

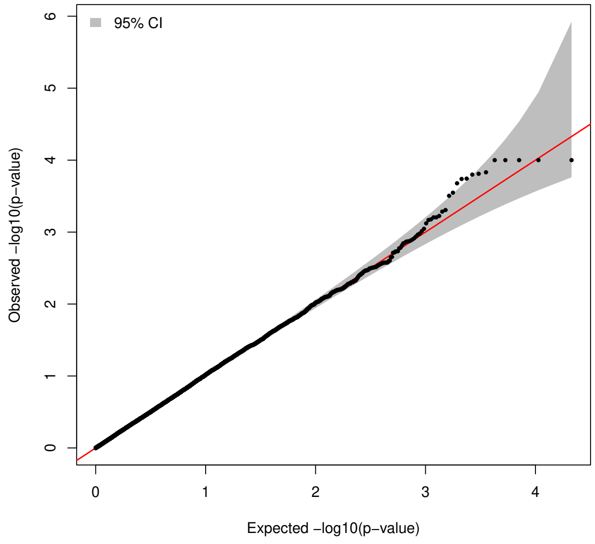

(a) IBS, S1

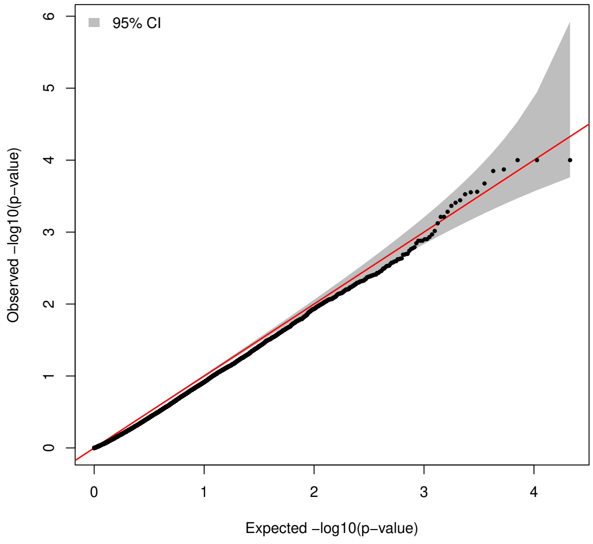

(b) IBS, S2

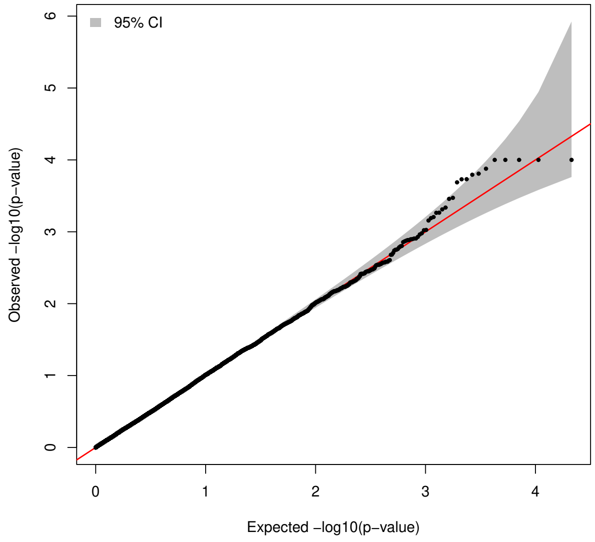

(c) IBS, S3

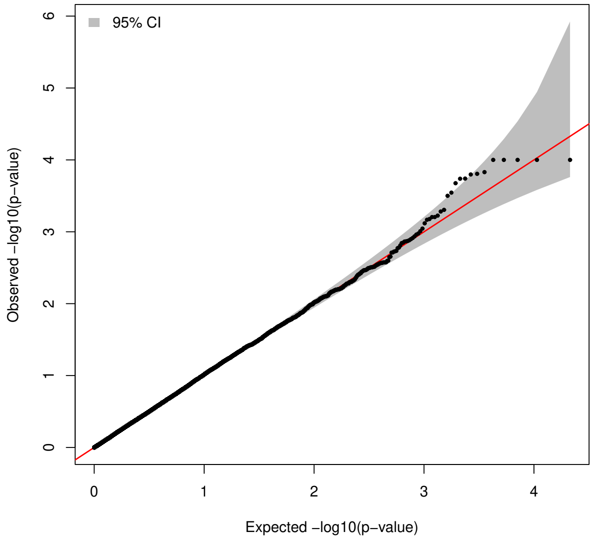

(d) IBS, S4

Web Figure 7. Genomic Q-Q plots of the p-value of R^c and R^c_{het} with the ROSMAP data. IBS genetic similarity kernel is used. Various types of heterogeneity were considered, including no genetic heterogeneity (S1), heterogeneity between sexes (S2), heterogeneity across education attainment categories (S3), and heterogeneity across genetic backgrounds (S4).

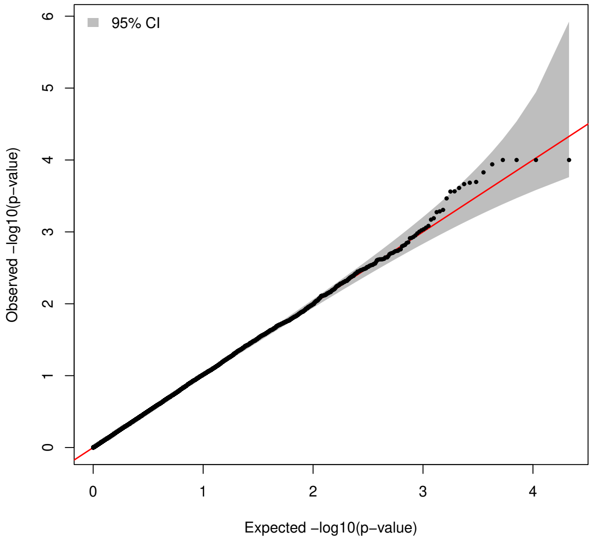

(a) Linear, S1

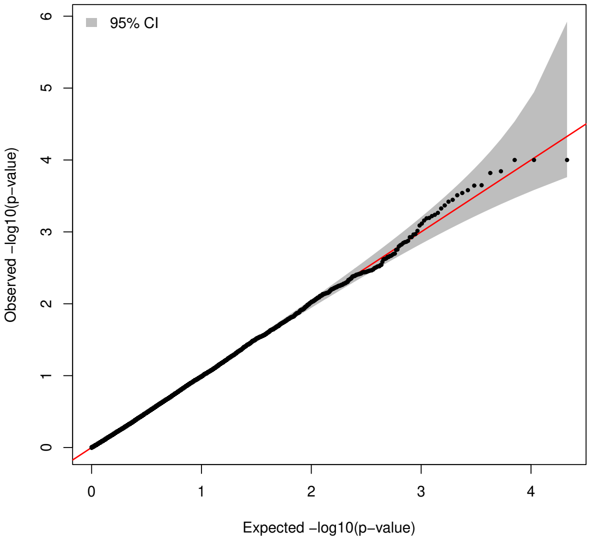

(b) Linear, S2

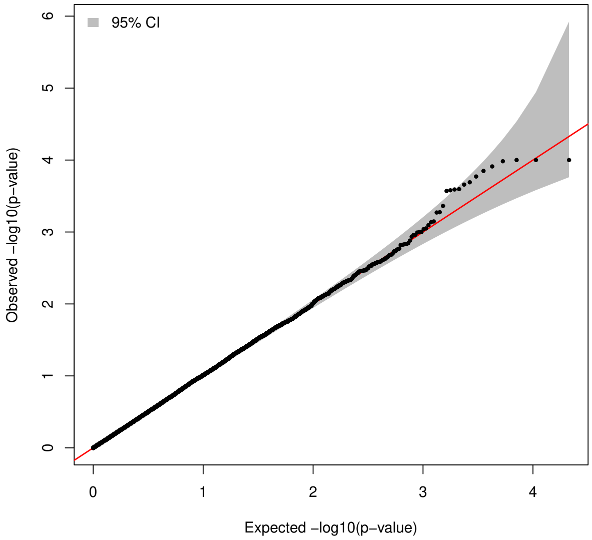

(c) Linear, S3

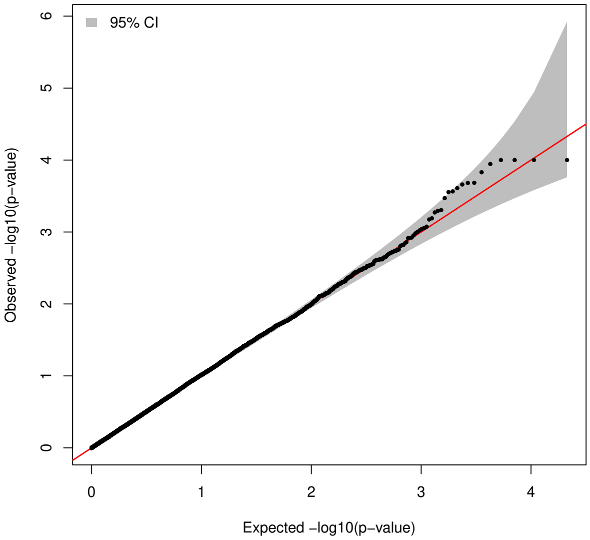

(d) Linear, S4

Web Figure 8. Genomic Q-Q plots of the p-value of R^c and R^c_{het} with the ROSMAP data. Linear genetic similarity kernel is used. Various types of heterogeneity were considered, including no genetic heterogeneity (S1), heterogeneity between sexes (S2), heterogeneity across education attainment categories (S3), and heterogeneity across genetic backgrounds (S4).

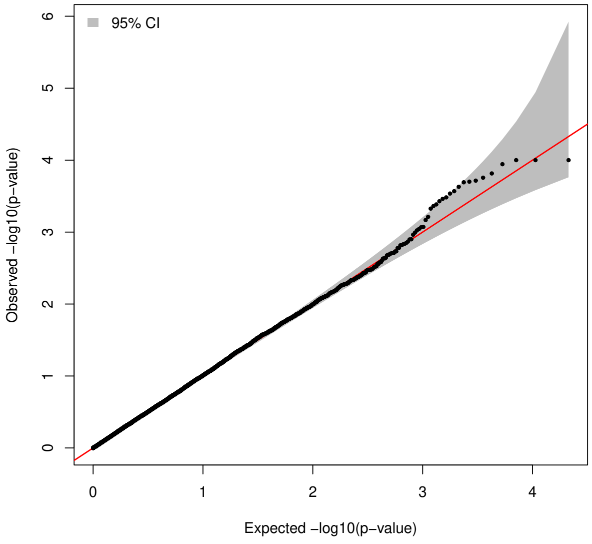

(a) Laplacian, S1

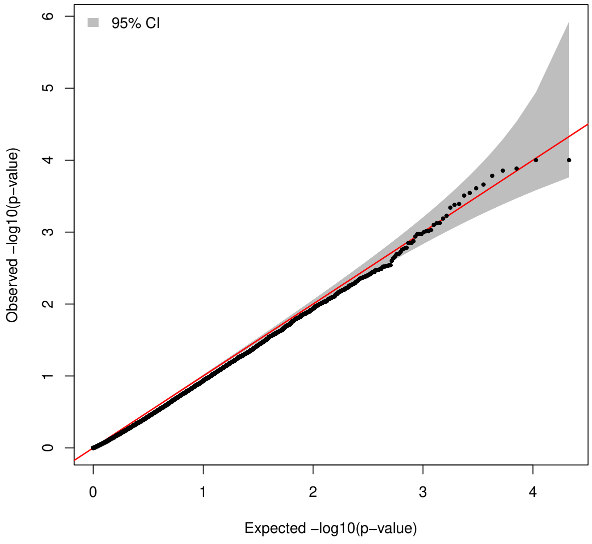

(b) Laplacian, S2

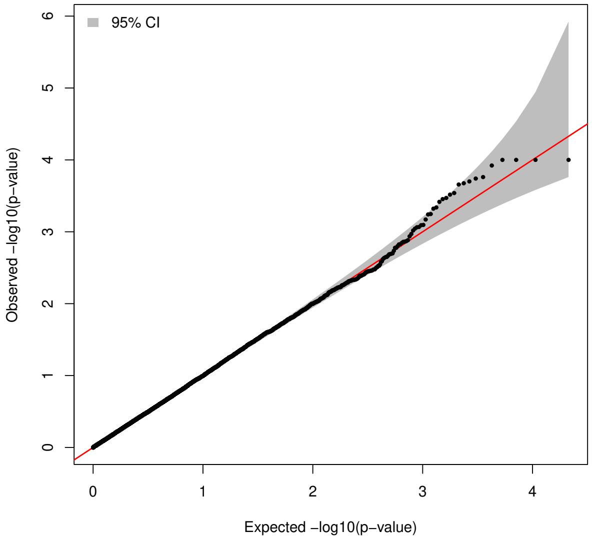

(c) Laplacian, S3

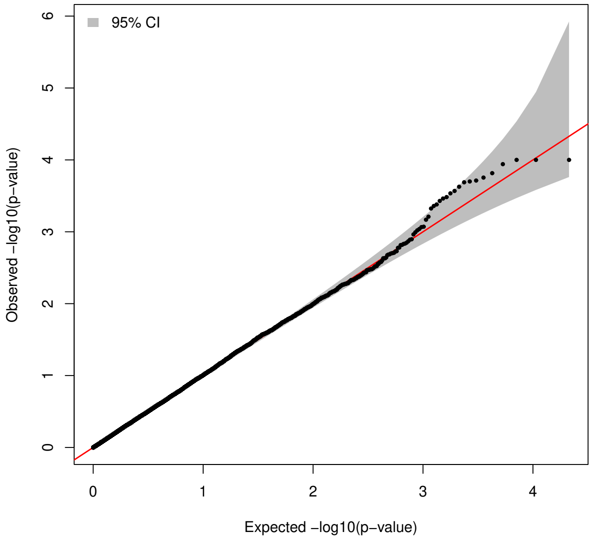

(d) Laplacian, S4

Web Figure 9. Genomic Q-Q plots of the p-value of R^c and R^c_{het} with the ROSMAP data. Laplacian genetic similarity kernel is used. Various types of heterogeneity were considered, including no genetic heterogeneity (S1), heterogeneity between sexes (S2), heterogeneity across education attainment categories (S3), and heterogeneity across genetic backgrounds (S4).

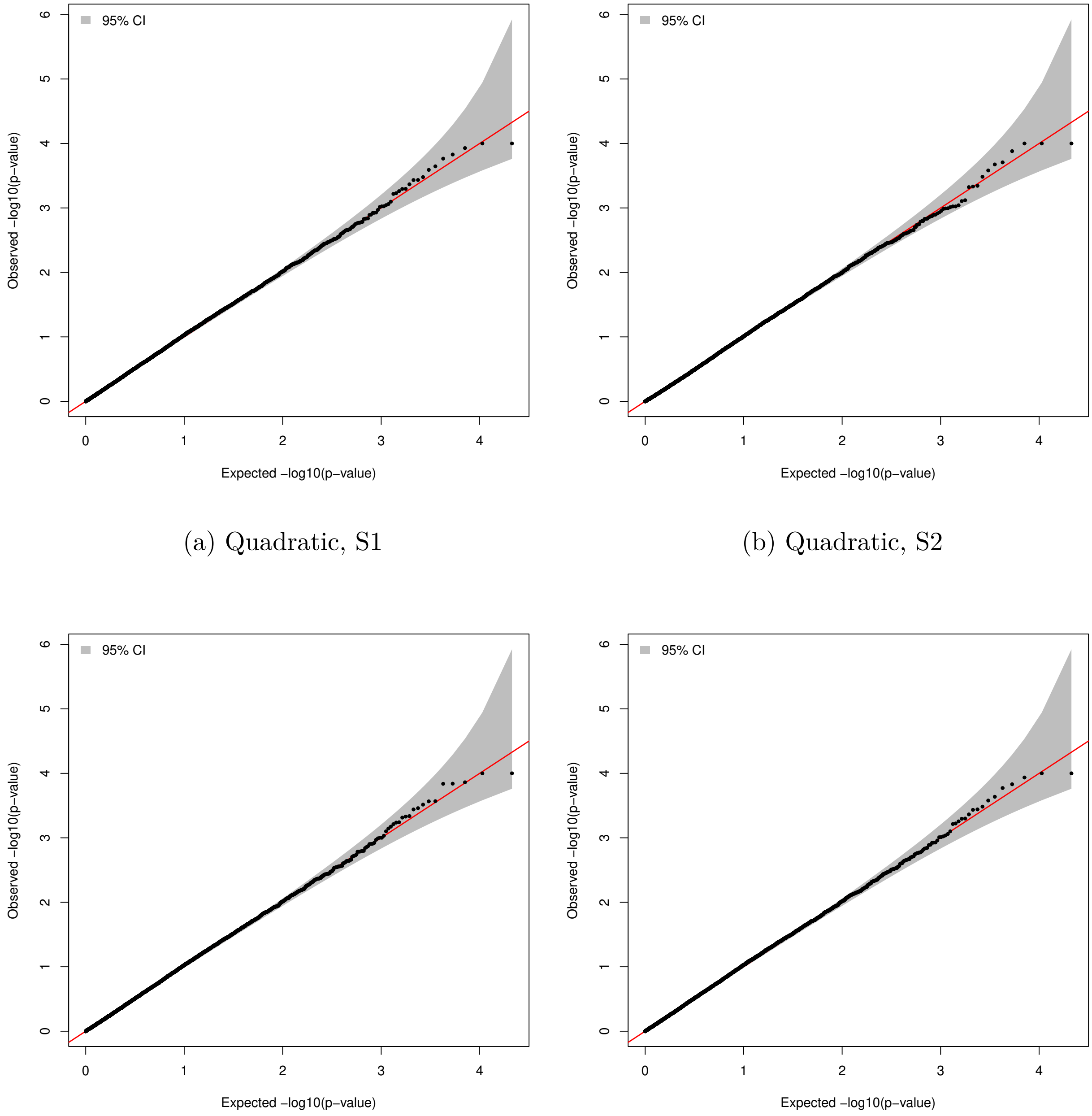

(a) Quadratic, S1

(b) Quadratic, S2

(c) Quadratic, S3

(d) Quadratic, S4

Web Figure 10. Genomic Q-Q plots of the p-value of R^c and R^c_{het} with the ROSMAP data. Quadratic genetic similarity kernel is used. Various types of heterogeneity were considered, including no genetic heterogeneity (S1), heterogeneity between sexes (S2), heterogeneity across education attainment categories (S3), and heterogeneity across genetic backgrounds (S4).
